# Hybrid Brain–Computer Interface Techniques for Improved Classification Accuracy and Increased Number of Commands: A Review

**DOI:** 10.3389/fnbot.2017.00035

**Published:** 2017-07-24

**Authors:** Keum-Shik Hong, Muhammad Jawad Khan

**Affiliations:** ^1^School of Mechanical Engineering, Pusan National University, Busan, South Korea; ^2^Department of Cogno-Mechatronics Engineering, Pusan National University, Busan, South Korea

**Keywords:** hybrid brain–computer interface, functional near infrared spectroscopy, electroencephalography, electrooculography, electromyography, classification accuracy

## Abstract

In this article, non-invasive hybrid brain–computer interface (hBCI) technologies for improving classification accuracy and increasing the number of commands are reviewed. Hybridization combining more than two modalities is a new trend in brain imaging and prosthesis control. Electroencephalography (EEG), due to its easy use and fast temporal resolution, is most widely utilized in combination with other brain/non-brain signal acquisition modalities, for instance, functional near infrared spectroscopy (fNIRS), electromyography (EMG), electrooculography (EOG), and eye tracker. Three main purposes of hybridization are to increase the number of control commands, improve classification accuracy and reduce the signal detection time. Currently, such combinations of EEG + fNIRS and EEG + EOG are most commonly employed. Four principal components (i.e., hardware, paradigm, classifiers, and features) relevant to accuracy improvement are discussed. In the case of brain signals, motor imagination/movement tasks are combined with cognitive tasks to increase active brain–computer interface (BCI) accuracy. Active and reactive tasks sometimes are combined: motor imagination with steady-state evoked visual potentials (SSVEP) and motor imagination with P300. In the case of reactive tasks, SSVEP is most widely combined with P300 to increase the number of commands. Passive BCIs, however, are rare. After discussing the hardware and strategies involved in the development of hBCI, the second part examines the approaches used to increase the number of control commands and to enhance classification accuracy. The future prospects and the extension of hBCI in real-time applications for daily life scenarios are provided.

## Introduction

Electroencephalography (EEG) and functional near infrared spectroscopy (fNIRS) endow brain–computer interfaces (BCIs) with their essential and indispensable attributes of non-invasiveness, low cost, and portability. EEG- and fNIRS-based BCIs have enabled paralyzed patients to communicate and control external devices with their own brain functions. Unfortunately, classification accuracy in these modalities diminishes as the number of BCI commands increases. As a mean of overcoming the problem of the reduction of classification accuracy upon an increase in the number of control commands, the concept of hybrid brain–computer interface (hBCI) was introduced (Allison et al., 2010; Muller-Putz et al., 2015; Banville and Falk, 2016).

The hBCI pursues the following three main objectives: (i) enhanced BCI classification accuracy, (ii) increased number of brain commands for control application, and (iii) shortened brain-command detection time. These benefits provide hBCI a clear advantage over any single brain signal acquisition modality. In this article, hBCI is meant to combine either (i) more than two modalities (of which at least one is a brain signal acquisition device) or (ii) more than two brain activities with a single modality, for example, the combination of P300 and steady-state visual evoked potential (SSVEP) with EEG (Allison et al., 2010; Pfurtscheller et al., 2010; Kreilinger et al., 2012; Muller-Putz et al., 2015).

First, classification accuracy can be improved by combining multiple signal features from different modalities/devices for the same brain activity. For example, EEG and fNIRS have been combined for the detection of finger tapping (Fazli et al., 2012) and hand/arm movement (Buccino et al., 2016). In these specific cases, the feature of EEG (i.e., signal band power) was combined with oxy- and deoxy hemoglobin (HbO and HbR) features of fNIRS to increase the accuracy of the system. Second, classification accuracy can be improved by utilizing one device’s signal in the artifact removal in another device’s brain signal. For instance, the peak value of electrooculography (EOG) caused by an eye blink (i.e., a motion artifact) can be subtracted from EEG’s data, in which the eye blink (or muscular movement) induces a false-positive value (McFarland and Wolpaw, 2011; Daly et al., 2015). The most common artifact removal means from brain signals are EOG (Bashashati et al., 2007; Jiang et al., 2014) and electromyography (EMG) (Fatourechi et al., 2007).

For proper operation of a BCI system, a certain number of control commands are required (Lafleur et al., 2013; Ramli et al., 2015). However, an increase in the number of commands in a BCI system will naturally diminish the classification accuracy (Vuckovic and Sepulveda, 2012; Naseer and Hong, 2015a). Hence, hBCI should have an advantage over a single modality in increasing the number of control commands without negatively impacting the accuracy. This is achieved by decoding multiple activities from different brain regions using different modalities. For instance, mental arithmetic (MA) tasks using fNIRS and motor-related tasks using EEG have been combined into an hBCI paradigm resulting in an improved classification accuracy (Khan et al., 2014). Researchers also tried to look for multiple brain regions to increase the number of commands. For example, SSVEPs were combined with event-related potentials (ERPs) to create a hybrid paradigm for EEG. A typical example is the combination of SSVEP with P300 signals for hBCI (Panicker et al., 2011; Li et al., 2013; Xu et al., 2013). Motor imagery (MI) also has been combined with SSVEP (Lim et al., 2015; Yu et al., 2015).

The detected brain signals can be categorized into three types (i.e., active, reactive, and passive) according to whether they were made intentionally, or reactively upon external stimulation, or unintentionally (Zander and Kothe, 2011). In the case of active BCI, an intentional brain task is used to generate the brain activity, for example, finger tapping, MA, MI, mental counting, and music imagery. In these tasks, a brain activity is generated objectively by the person without any external stimuli and hBCI can be made using the brain signals in association with the performed mental tasks (Power et al., 2010). In the case of reactive BCI, external stimuli are provided to cause a brain activity. In this paradigm, the stimuli can be given in various forms, for instance, audio (Santosa et al., 2014; Hong and Santosa, 2016), video (Li et al., 2013; Zhang et al., 2013, 2014), interrogative (Hu et al., 2012; Bhutta et al., 2015), and pain (Hong and Nguyen, 2014). The hBCI combining SSVEP and P300 in EEG is considered reactive. In the case of passive BCI, an arbitrary brain signal generated by the subject with no intention—for instance, a signal related to drowsiness, vigilance, and fatigue—can be used (Khan and Hong, 2015). With regard to drowsiness, EEG and EOG are simultaneously checked to create an hBCI paradigm for accident avoidance (Picot et al., 2012).

Herein, we present a review of the various hBCI technologies. The schemes of non-invasive methodology in enhancing the BCI accuracy are discussed first. Note, however, that the studies combining only features and algorithms to decode activities for a single modality are excluded. Also, the hybrid systems that are not specifically used for BCI are excluded.

**Figure 1:**
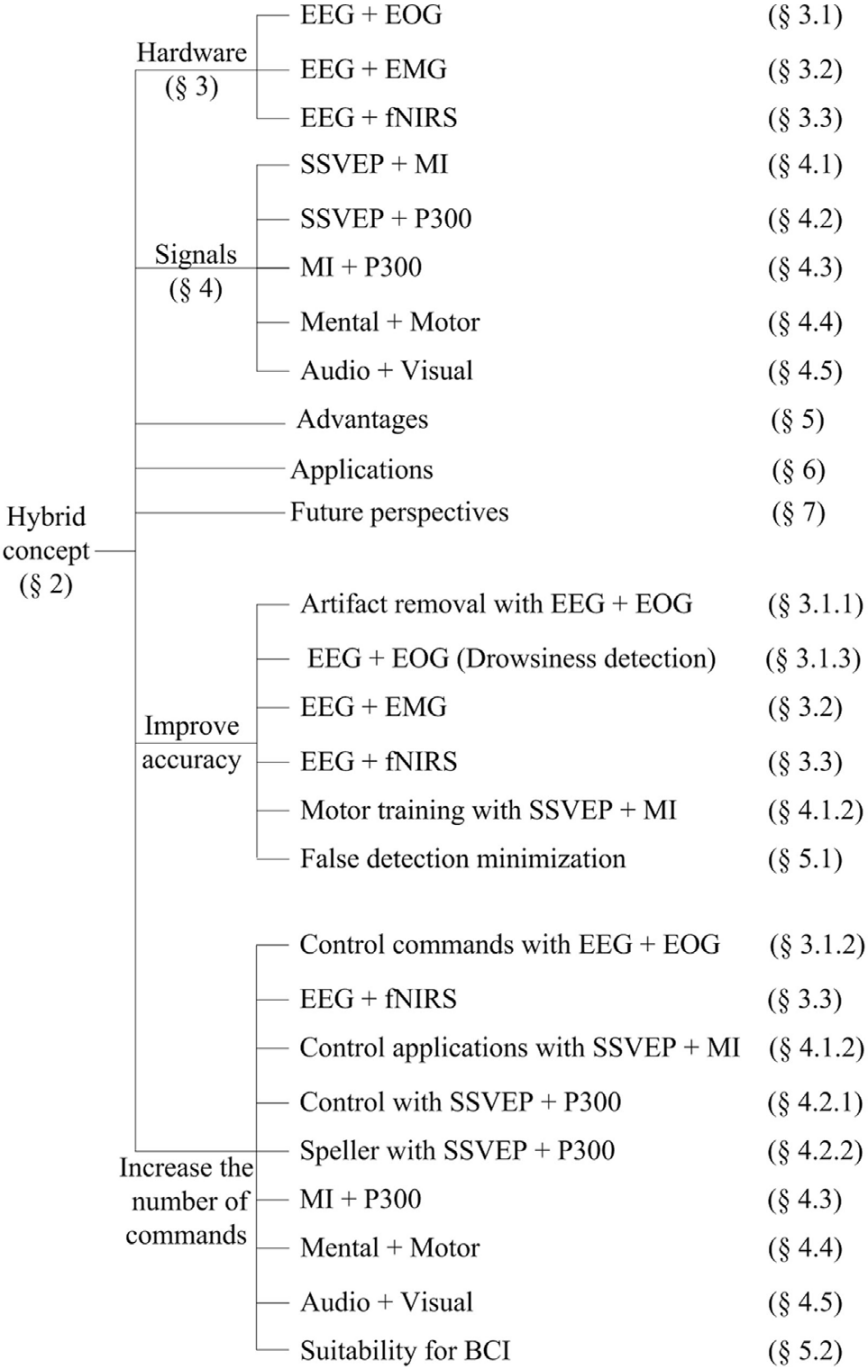
Breakdowns of the paper.

Figure 1 breakdowns the contents of the entire paper. The first part of this article introduces the concept of hybrid system, which utilizes a combination of different hardware to enhance BCI accuracy and to increase the number commands. Section “Hardware Combination” describes different combinations of hardware appeared in the literature. Section “Combination of Brain Signals” evaluates the combination of brain signals decoded by a single brain signal acquisition modality. Section “Advantages of hBCI” discusses the applications of hBCI systems for healthy people as well as patients. Section “Applications” explains the advantages of hybrid systems over single-modality versions. This section also provides detailed tables on hBCIs in terms of active, reactive, and passive tasks. The last part of this article discusses the future prospects for, and the research directions of, hybrid systems in control and rehabilitation applications.

## Hybrid Concept

Pfurtscheller et al. (2010) explained that an hBCI system is similar to a simple BCI but that it needs additionally to fulfill the following four criteria: (i) the activity should be directly acquired from the brain; (ii) at least one of multiple brain signal acquisition modalities should be employed in acquiring such activity, which can be in electrical potential, magnetic field, or hemodynamic change form; (iii) the signals must be processed in real time/online to establish communication between the brain and a computer for generation of control commands; and (iv) feedback describing the outcomes of the brain activity for communication and control must be provided.

Recent hBCIs based on these criteria have focused on improving the accuracy of activity detection and increasing the number of control commands to achieve better communication and control for healthy subjects as well as patients. This is especially true considering the fact that an hBCI consists of at least two modalities (one of which is a brain-based signal) working in concert with each other to produce better BCI functionality.

Six aspects (hardware, signal processing, brain activity, feature extraction, classification, and feedback) need to be considered in developing an hBCI: (i) the hardware should consist of at least one brain signal acquisition modality; (ii) the hybrid system should detect and process different physiological signals simultaneously; (iii) the paradigm should be able to acquire multiple brain activities simultaneously using multiple modalities; (iv) a number of features for classification should be acquired in real time/online for both accuracy enhancement and additional control-command generation; (v) the classified output should have a potential for interfacing with external devices (e.g., wheelchairs and robots); and (vi) it should also provide feedback to the user for rehabilitation and control purposes (Nicolas-Alonso and Gomez-Gil, 2012; Ramadan and Vasilakos, 2017).

**Figure 2:**
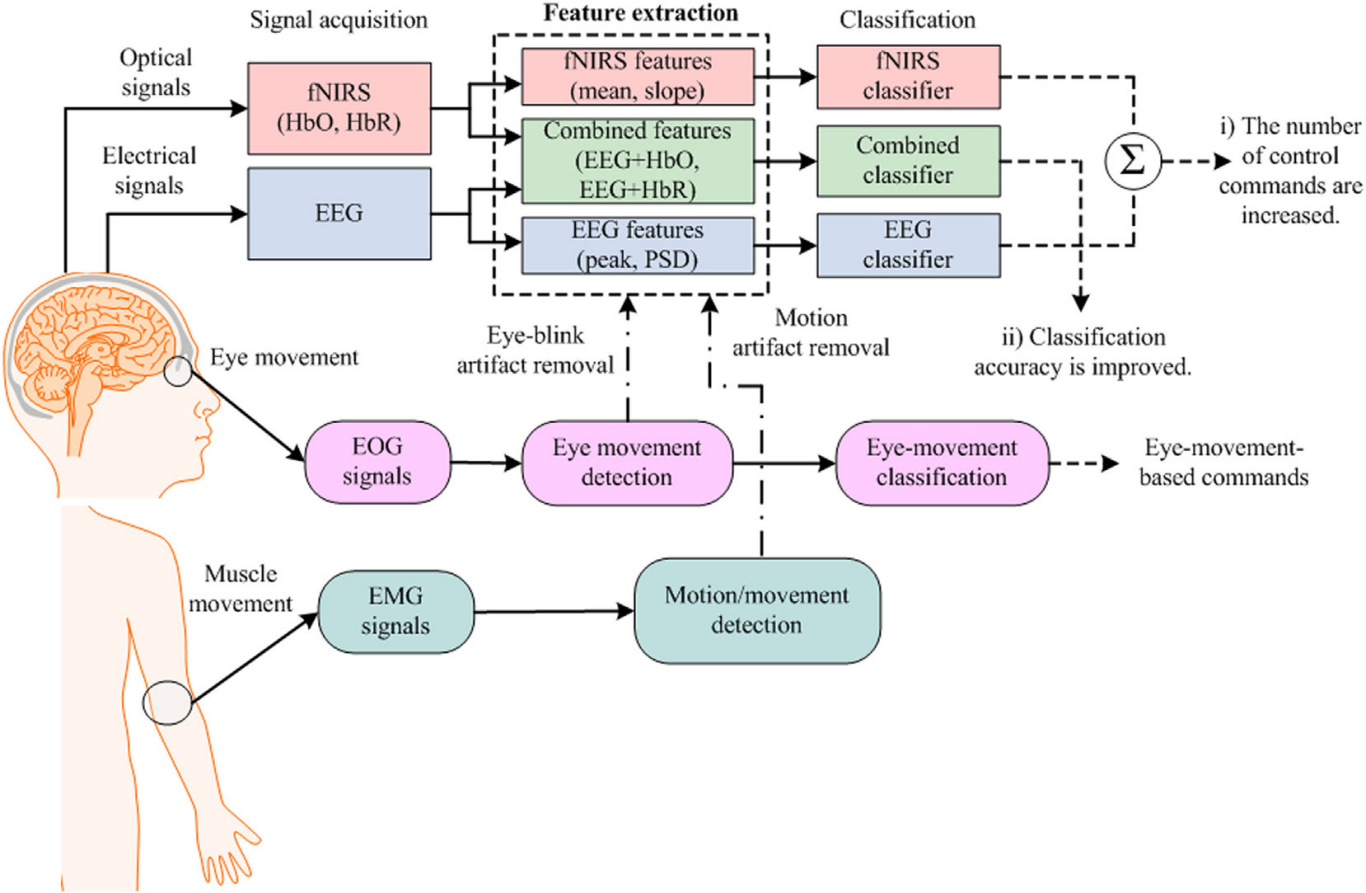
Purposes of hybrid brain–computer interface: (i) increase the number of control commands by combining electroencephalography (EEG) with functional near infrared spectroscopy (fNIRS) [further electrooculography (EOG)] and (ii) improve the classification accuracy by removing motion artifacts.

Figure 2 provides an example of an hBCI scheme. It indicates the following two things: (i) multiple activities are required for hBCI and (ii) a combination of brain and non-brain signal acquisition modalities is overviewed. After detection, the activities are processed simultaneously for feature extraction and classification; then, the classified results are used as feedback for the user’s rehabilitation and control applications.

## Hardware Combination

Hybrid brain–computer interface hardware can be configured in the following two ways: (i) combination of a brain signal acquisition modality with a non-brain signal acquisition modality (Fatourechi et al., 2007; Li et al., 2015; Yang et al., 2015) and (ii) combination of a brain signal acquisition modality with another brain signal acquisition modality (Kaiser et al., 2014; Putze et al., 2014). Brain and non-brain signal acquisition modalities are combined either to remove motion artifacts or to increase the number of commands in a BCI system. Two brain signal acquisition modalities are combined and positioned over the same brain region in order to enhance the classification accuracy, or, they are positioned in different regions to increase the number of control commands. In the case of portable devices, the following signals are used.

### Neuronal Signals

These are measured as a difference in voltage between two different cerebral locations over time. The signal is recorded by EEG electrodes positioned on the scalp. The recorded potential difference is reflected as the postsynaptic potential in the cell membranes of cortical neurons (Olejniczak, 2006; Nguyen and Hong, 2013). These signals are most effective for BCI, as they are detected immediately (e.g., P300 signals are detected 300 ms after stimuli are given). These signals also contribute in the detection of brain drowsiness state (Qian et al., 2016, 2017).

### Hemodynamic Signals

The hemodynamic response is a process in which the blood releases glucose to active neurons at a greater rate than inactive ones. The glucose with oxygen delivered through the blood stream results in a surplus of HbO in the veins of the active area as well as a distinguishable change of the ratio of local HbO to HbR. These changes are detected by functional magnetic resonance imaging (fMRI) and fNIRS (Boas et al., 1994, 2001, 2014; Huppert et al., 2009, 2013; Nicolas-Alonso and Gomez-Gil, 2012). These signals have an inherent delay in hemodynamic response generation. However, the most recent discovery on initial dips makes the HbO signals a viable candidate for BCI (Hong and Naseer, 2016; Zafar and Hong, 2017).

### Eye Blink/Eye Movement Signals

The eye can be modeled as a dipole, with its positive pole at the cornea and its negative pole at the retina. Assuming a stable corneo-retinal potential difference, the eye is the origin of a steady electric potential field. The electrical signals generated from this field are measured by EOG (Bulling et al., 2011). Sometimes an eye tracker also is used for the detection of eye movements. Mostly, these signals are used for the investigation of vigilance and drowsiness activities.

### EMG Signals

These signals are an indication of muscles’ electrical activity, which arises whenever there exists a voluntary or involuntary contraction (Chowdhury et al., 2013; Patil and Patil, 2014; Xie et al., 2014). These signals are recorded by EMG electrodes, which are most widely used in neuro-prostheses (Ravindra and Castellini, 2014; Chadwell et al., 2016; Chen et al., 2016). Table 1 summarizes possible combinations that can be used in the development of hBCI hardware.

**Table 1:** Combinations of devices.

Modality combination	Sensor placement	Signal combination	Possible outcome
Electroencephalography (EEG) + electrooculography (EOG)	Brain and eyes	Electrophysiological + eye movement	Increase in control commands/increase in accuracy
EEG + electromyography (EMG)	Brain and muscles	Electrophysiological + electromyography	Increase in accuracy
EEG + functional near infrared spectroscopy (fNIRS)	Brain	Electrophysiological + hemodynamic	Increase in classification accuracy/increase in control commands

### EEG + EOG

**Figure 3:**
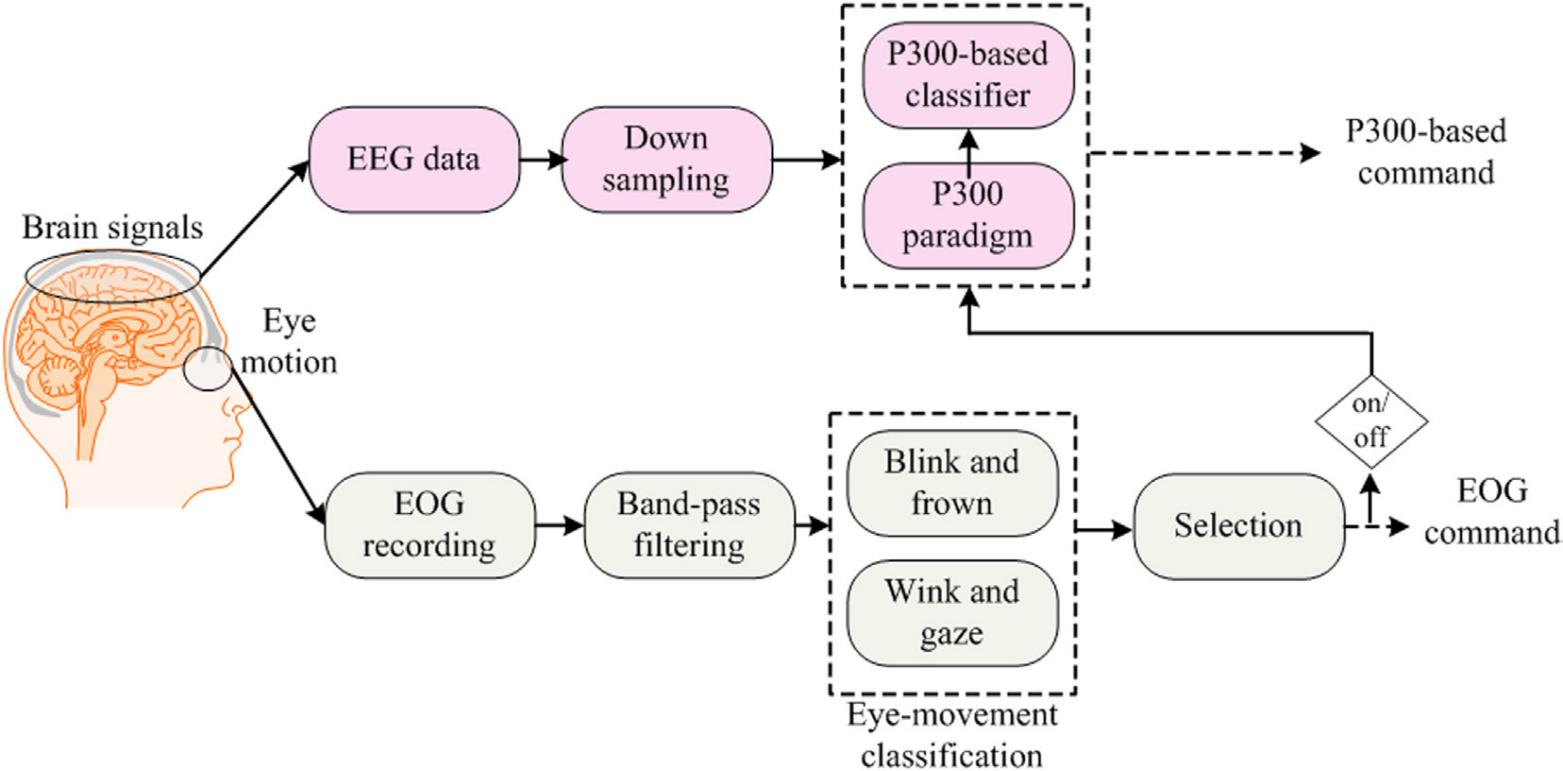
Electroencephalography (EEG)–electrooculography (EOG)-based brain–computer interface: the blink signals are used for switching between EEG- and EOG-based command generation, in which EEG and EOG generate P300-based commands and frown–wink–gaze-based commands, respectively.

EOG-based BCIs are useful for people who have control over their eye movements, as by this means, multiple commands can simultaneously be generated. Combinations of eye movement signals (blink, wink, frown, etc.) with neuronal signals usually are utilized for hybrid EEG–EOG-based BCIs (Ma et al., 2015). In this section, we also include hybrid studies that have used eye-tracking with EEG to develop hybrid systems for BCI. We discuss EOG and eye tracker-based studies together, as both use eye movements for classification. For command generation, signals are decoded simultaneously, and for control of a BCI system, they are fused using a combined classifier (Jiang et al., 2014). Although EOG is used to remove ocular artifacts from EEG data (Li et al., 2015), drowsiness detection (Khushaba et al., 2011) and wheelchair control (Ramli et al., 2015) are also among the most common applications of EEG–EOG-based systems. Figure 3 shows the method used to acquire simultaneous EEG and EOG data for analysis.

#### Artifact Removal

Eye blink signals influence brain signals by inducing artifacts in the data. Due to such ocular artifacts, false-positive signals appear in EEG data, which leads to misclassification and false-command generation (Trejo et al., 2006).

A pioneering study in Bashashati et al. (2007) tested the performance of EEG-based self-paced BCI by investigating the effects of eye blink signals on data. The results showed that the removal of eye blink signals improves BCI performance. Another study (Fatourechi et al., 2007) reviewed ocular artifacts in EEG data and proposed that the EEG–EOG combination might result in better output than an individual modality. Hsu et al. (2012) performed a single-trial classification to evaluate accuracy differences between artifact-removed and non-artifact-removed data. Linear discriminant analysis (LDA) and support vector machine (SVM) have been used to classify the data obtained from the motor cortex region. The results showed that the average classification accuracy obtained by removing EOG artifacts was higher than that from non-artifact removal data. Using artifact-removed features, the obtained accuracies were 84.4% for both LDA and SVM, whereas using the non-artifact-removed features, only 80.9 and 77.7% accuracies were achieved. This study reveals that, with EEG data, EOG artifacts have a decremental effect on classification accuracy. In similar studies, an automatic artifact correction that combines a regression analysis has been successfully implemented for MI tasks (Wang et al., 2012). Also, entailing the removal of EOG signals (using eye tracking), thresholding has been reported to increase classification accuracy [usingstep-wise LDA (SW-LDA)] from 44.7 to 73.1% in hBCI (Yong et al., 2012). Independent component analysis (ICA), genetic algorithm (GA), and particle swarm optimization for EOG artifact detection and removal also have been reported in the literature (Hsu, 2013a,b; Daly et al., 2015; Li et al., 2015; Yang et al., 2015). Bai et al. (2016) has recently proposed an ICA-based method to reduce the muscular/blink artifacts appearing in the prefrontal cortex after brain stimulation. The eye movement and muscle artifacts were detected using EEG. Ensemble empirical mode decomposition was used to decompose signal into multi-components, and then, the components were separated with artifact reduced by blind source separation method.

#### Control Commands

The combination of EEG and EOG is important to the improvement of the classification accuracy of BCI systems by artifact removal (Zhang et al., 2010). This combination can also be used to increase the number of control commands. For this type of hBCI, eye blink and eye movement signals are used for command generation (Roy et al., 2014; Belkacem et al., 2015b).

Among such applications, early studies have proposed the control of wheelchairs using EEG and EOG signals (Kim et al., 2006). This initial work used the hidden Markov model (HMM) to obtain an accuracy of 97.2% for wheelchair control. Eye gaze signals were later used to implement wheelchair control using SVM as a classifier, in which case, an accuracy of above 80% was achieved (Lamti et al., 2013). In another work, eyeball and eyelid movements were detected using EEG for wheelchair control (Aziz et al., 2014) and the features were extracted using eye opening, eye closing, and eye gaze directionality, thus achieving a 98% accuracy using HMM as a classifier. MI, P300, and eye blinking have also been applied for control of a wheelchair in four directions using SVM-based classification (Wang et al., 2014), thereby obtaining an average accuracy of above 85%. In a similar work on hBCI, eye gaze and EEG signals were trained and tested for wheelchair control using a finite-state machine and neural-network-based classifier and six control commands were generated, achieving an accuracy of 97.8% (Ramli et al., 2015). Among other works on EEG–EOG-based BCI, 2D cursor control has been implemented, using kernel partial least square classification, with accuracies ranging between 80 and 100% (Trejo et al., 2006). EOG and EMG have also been combined with EEG to improve mental task classification using Fisher discriminant analysis (Zhang et al., 2010). Another study of Jiang et al. (2014) has shown that features selected based on different eye movement and gaze signals led to 89.3% accuracy using LDA as a classifier. Real-time video game control of Belkacem et al. (2015a) and exoskeleton control of Witkowski et al. (2014) have also been implemented in the form of hBCI using a thresholding scheme with the accuracies of 77.3 (for six commands) and 63.59% (for four commands), respectively. Additionally, robotic arm control (Hortal et al., 2015), mobile robot control (Ma et al., 2015), and quadcopter control (with eye tracking) (Kim et al., 2014) have been developed using EEG–EOG-based hBCI. Most recently, a gaze-based game using intentional and spontaneous eye movements with EEG as a marker to control was developed (Shishkin et al., 2016): classification of intentional VS spontaneous fixations was based on amplitude features from 13 EEG channels using 300 ms moving window. A 300 ms EOG moving window was used to remove the eye movement-related artifacts from the data. For the first fixations in the fixation triplets required to make moves in the game, LDA-based classification was used to achieve 90% accurate results. Another interesting study has demonstrated the movement control of a turtle using human brain signals (Kim et al., 2016). In this case, SSVEP-based tasks were combined with an eye-tracking device to control the turtle in real time. The system consists of a glass involving two flickering signals for SSVEP generation and direction arrows for detection using eye tracking. The movement commands were generated using canonical correlation analysis (CCA) in a 2 s window. Four commands were generated using the scheme in which two (turn left/right movements) commands were generated using SSVEP and other two commands (reset and idle) using eye tracking based on eye opening and closing. The circuit implanted in the brain of the turtle controlled its motion using human brain signals *via* Wi-Fi communication. The event-related desynchronization and SSVEP accuracies achieved were 88.3 and 92.7%, respectively.

#### Drowsiness Detection

Several EEG and EOG studies have investigated the detection of drowsiness (Dorokhov, 2003; Duta et al., 2004; Papadelis et al., 2007; Virkkala et al., 2007a,b). Different feature extraction algorithms have shown the effectiveness of individual EEG system for drowsiness detection (Qian et al., 2016, 2017), and the combination of EEG and EOG seems to be more effective (Sinha, 2008; Gharagozlou et al., 2015; Khemiri et al., 2015). For combined EEG–EOG signals, various strategies have been adopted. Among the recent works since 2010, fuzzy mutual-information-based wavelet transform has been used for drowsiness detection to a high (95–97%) accuracy (Khushaba et al., 2011). EOG signals and visual information have been utilized in the generation of warnings for drowsy drivers (Picot et al., 2012; Akerstedt et al., 2013). Maximum overlap wavelet transform has been implemented with an accuracy of 96.06% to detect various stages of sleep (Khalighi et al., 2013). Fuzzy neighborhood-preserving analysis showed a 93% accuracy for drowsiness detection (Khushaba et al., 2013), and a neural-network-based extreme learning algorithm obtained 95.6% accurate results for alertness and drowsiness signals (Chen et al., 2015). The most recent work on drowsiness/vigilance estimation using real-time brain monitoring has achieved 80.4% accurate results by combining EEG and EOG (Cao et al., 2016), in which the EEG bands (α, β, θ, and *Δ*) were combined together with EOG using LDA-based classification to develop a real-time drowsiness detection system for drivers.

### EEG + EMG

**Figure 4:**
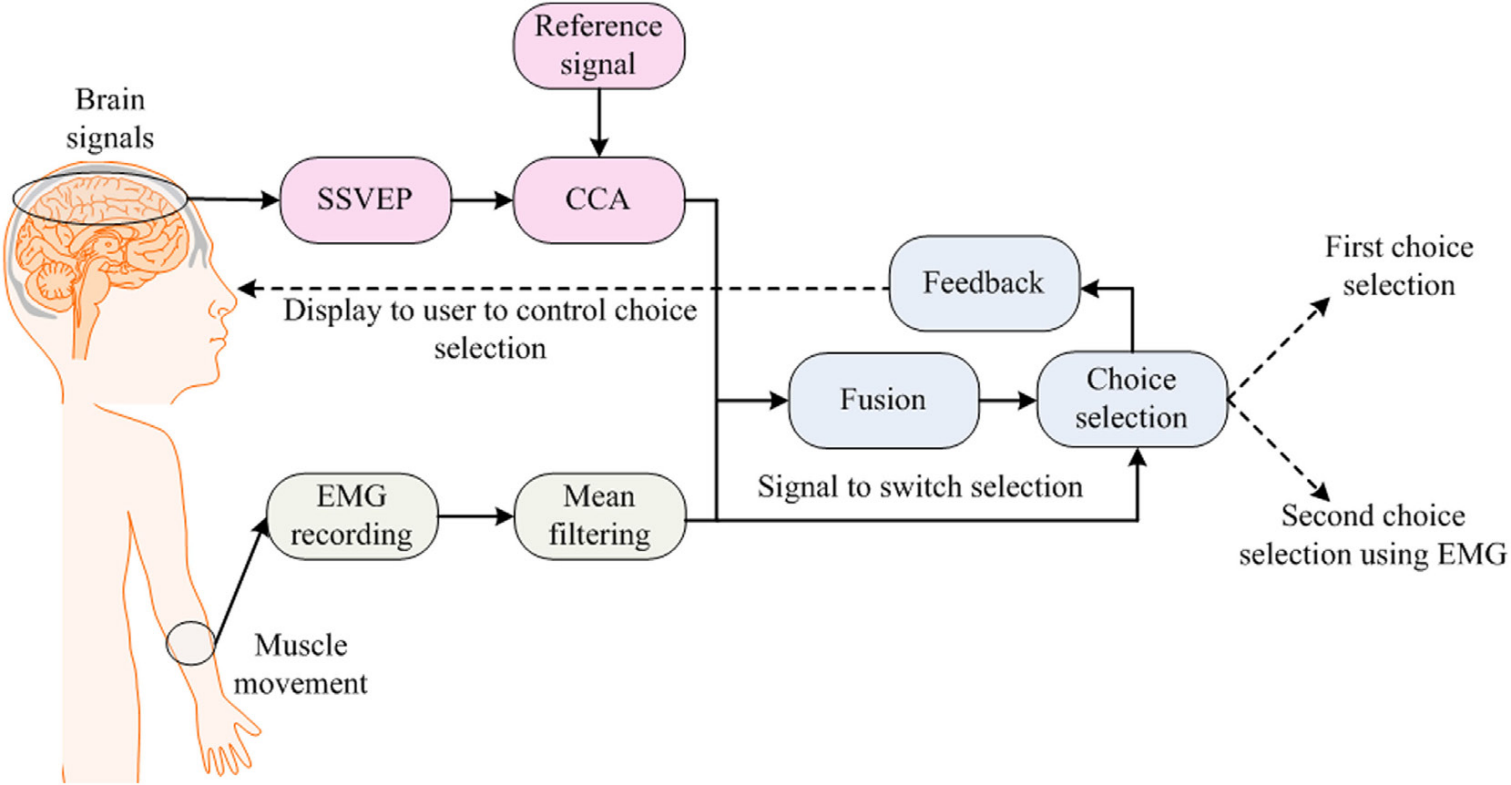
Electroencephalography–electromyography (EMG)-based brain–computer interface: one choice is selected using steady-state visual evoked potential (SSVEP) and muscle movement is used to change the selected option.

Electromyography signals are generated and detected as a result of muscular movement (Trejo et al., 2003; Foldes and Taylor, 2010; Cler and Stepp, 2015). These act as an artifact in EEG signals, resulting in the false detection of brain signals (Fatourechi et al., 2007; Bhattacharyya et al., 2013). The purpose behind a hybrid EEG–EMG-based hBCI is to combine EEG and EMG signals in hBCI. This incorporation of EMG signals is user specific and depends on the activity or task performed by that user. The applications of hybrid approaches vary from a simple game control application for an able-bodied person through to a prosthetic arm control application for an amputee. Figure 4 shows a typical strategy used for incorporating EEG and EMG signals into an hBCI system.

The applications of EEG–EMG-based hBCI are found in the control area of assistive devices (Leeb et al., 2011; Kiguchi et al., 2013). In the early work using EEG with EOG and EMG (Kennedy and Adams, 2003), the EMG signals were used to categorize different “locked-in” patient types. In their study, six types were defined, the first three of which were categorized using EMG as follows:

- Patients capable of movement (e.g., eye movement and finger movement).
- Patients incapable of movement but showing some detectable EMG activity due to partial muscle movements.
- Fully locked-in patients with no muscular activity detectable by EMG signals.

The remaining three types of patients were categorized using EOG and EEG signals. For EEG–EMG-based BCI, a neuro-electric interface was developed for real-time applications (Trejo et al., 2003). In 2005, a BCI that removes EMG artifacts from EEG for mouse-cursor control was developed (McFarland et al., 2005). In 2007, a detailed survey on EMG artifacts in EEG signals was presented (Fatourechi et al., 2007). In 2010, a study by Brumberg et al. (2010) combined EEG and EMG for tetraplegic patients: their results showed that communication, however slow, can be achieved using EEG–EMG-based hBCI. In that same year, jaw muscle contraction and EEG signals were used to generate commands for an assistive neuro-prosthetic device (Foldes and Taylor, 2010). Also, the use of EMG with EEG was explored in a review article on the operation of robotic and prosthetic devices (McFarland and Wolpaw, 2010). In 2011, an investigation was conducted for the prediction of voluntary movements before their occurrence using hBCI (Bai et al., 2011). Vehicle steering (Gomez-Gil et al., 2011) and determination of muscle fatigue levels (Leeb et al., 2011) using EEG–EMG-based BCI have also been reported in the literature. In 2012, simultaneous measurement of EEG–EMG signals led to the achievement of an assistive control of exoskeletons for locomotion (Cheron et al., 2012), wherein a surface-tactile stimulation device was used for the training of brain signals, and dynamic recurrent neural network-based classifiers were used for training and testing of brain signals. Single-trial decoding of reaching movement also was conducted using EEG–EMG-based signals (Demandt et al., 2012). A similar study (Kiguchi et al., 2013) in 2013 proposed EEG–EMG-based control of an artificial arm for above-elbow amputees. An EEG–EMG-based motion estimation method was proposed for the control of the forearm and the supination/pronation motion of the artificial arm. In 2014, signals produced by jaw clenching were removed from EEG signals for two-dimensional cursor control on a computer screen (Costa et al., 2014). This study was later extended to the control of a robotic arm in bi-dimensional workspace. In 2015, EMG was used in rehabilitation applications for robot-assisted exercise tasks (Fels et al., 2015). In this case, neuro-feedback was used for intensive motor training and EEG–EMG was employed to predict the workload profiles for the experience of frustration. A review article by Rupp et al. (2015) also discussed the application of EMG for a hybrid neuro-prosthesis, proposing the use of functional electrical stimulation (FES) for therapy. The scheme used EEG–EMG to record brain activity and to investigate, on that basis, recovery in muscles and the brain. Most recently, new work has been done, which combines SSVEP-based tasks with EMG for choice selection (Lin et al., 2016). A 60-target hybrid BCI speller was built in this study. A single trial was divided into the following two stages: a stimulation stage and an output-selection stage. In the stimulation stage, SSVEP and EMG were used together. Every stimulus flickered at its given frequency to elicit SSVEP. CCA and mean filtering were used to classify SSVEP and EMG, respectively. In the result, 81% of accurate results were obtained by hybridizing EMG with SSVEP activities.

### EEG + fNIRS

**Figure 5:**
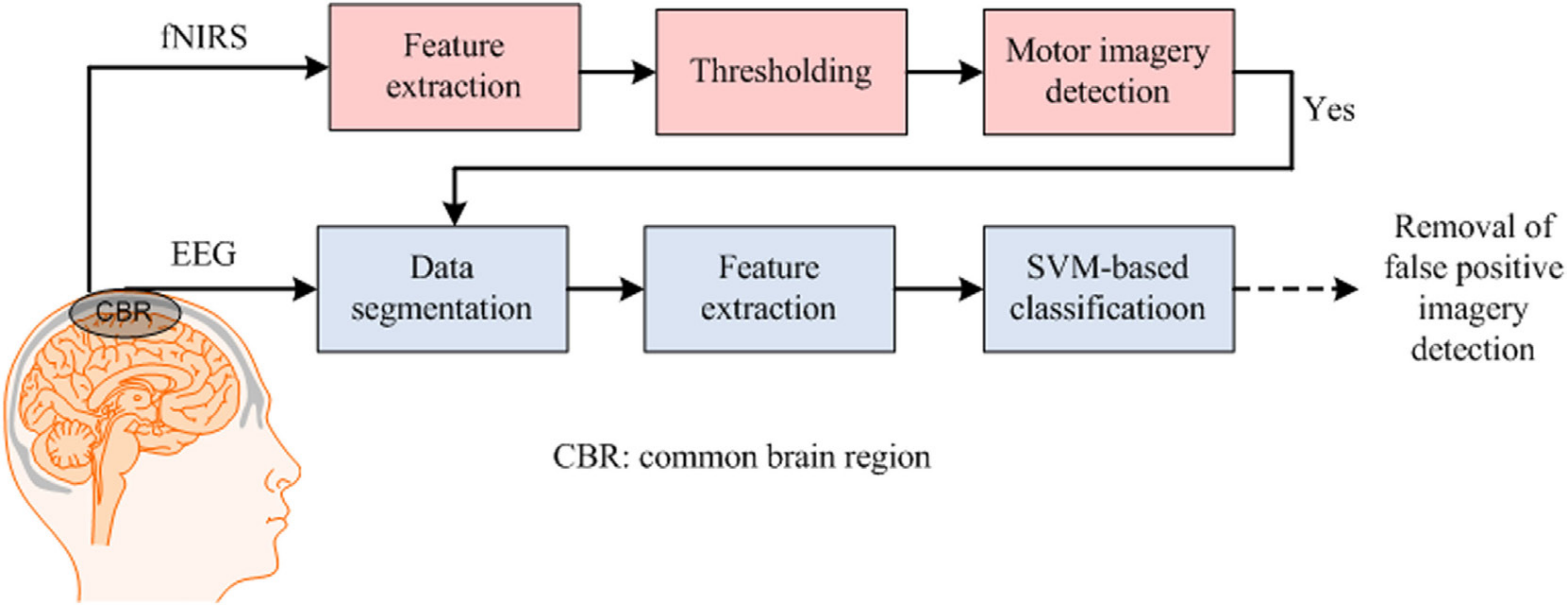
Electroencephalography (EEG)-NIRS-based brain–computer interface: the figure shows a method of removal of false-positive motor imagery signals in EEG data using functional near infrared spectroscopy (fNIRS) (delayed decision).

The research completed on hybrid EEG–fNIRS is still very limited. This technology is used mostly to improve classification accuracy (Fazli et al., 2012) or increase the number of control commands (Khan et al., 2014) in a BCI system. Although the research has shown good results for the combination of fNIRS with bio-signals (Zimmermann et al., 2013), hybrid EEG–NIRS has shown the best results thus far for BCI. In this case, two brain signal acquisition modalities are combined using neuronal signals (recorded using EEG) and hemodynamic signals (recorded using NIRS). One important disadvantage of the use of hemodynamics (either fMRI or fNIRS), however, is the inherent delay in the response (Huppert et al., 2013), which renders the generation of commands slow in comparison to EEG. However, in the case of combined EEG–fNIRS, this kind of disadvantage can be removed. Also, the detection of initial dip (i.e., the phenomenon that HbO decreases and HbR increases with neural firing) instead of hemodynamics might lead to a better time window selection for the combined modalities. Figure 5 shows an approach used to combine the EEG–NIRS modalities for BCI.

The first study on hybrid EEG–NIRS for application to BCI appeared in 2012 (Fazli et al., 2012). It showed that the combination of fNIRS’s features (HbO and HbR) and EEG features increases the classification accuracy. In this case, a multi-class classifier that combined the NIRS and EEG features for classification was used. The results showed that using EEG + HbR features, the average classification accuracy for motor execution tasks was improved from 90.8 to 93.2%. Similarly, for MI tasks, the average classification accuracy using EEG + HbO features was increased from 78.2 to 83.2%.

In 2013, a study of Safaie et al. (2013) analyzed the steps involved in the development of hardware for a hybrid EEG–NIRS system, among which was the design of a wireless wearable module for simultaneous decoding of brain activity. In 2014, an optimal time window for hybrid EEG–NIRS features selection was investigated using a SSVEP-based paradigm (Tomita et al., 2014). The results showed that the optimal window for EEG and fNIRS is 10 s. Another study by Khan et al. (2014) showed that the number of control commands can be increased by simultaneously decoding the EEG and fNIRS activities from different brain locations, in which LDA was used as a classifier for both EEG and fNIRS, MA and mental-counting tasks were decoded using fNIRS, and left- and right-hand tapping were coded using EEG. An offline study on tetraplegia patients (Blokland et al., 2014) showed that combined EEG–NIRS can be used to decode motor attempts and imagined movement with high accuracies up to 87 and 79%, respectively. A logistic regression classifier based on the Bayesian theorem was used for classification, specifically by obtaining the EEG data in a 0–15 s window and the fNIRS data for classification in a 3–18 s window. The study showed that the highest accuracy for tetraplegia patients was obtained by combining EEG with HbR. Kaiser et al. (2014) investigated the effect of training on cortical brain areas using EEG–NIRS with MI as a task. The study compared the subjects with high BCI performance (accuracy > 70%) with those with low BCI performance (accuracy < 70%), employing LDA for acquisition of the classification accuracies. The results showed that training with MI-based BCI affects cortical activations, especially with those subjects showing a low BCI performance. In another work, hybrid EEG–NIRS showed higher classification accuracies in discriminating auditory and visual stimuli (Putze et al., 2014). In this work, SVM was used as a classifier to discriminate the visual and audio cues and, thus, to develop an hBCI; the accuracy achieved was as high as 94.6%.

In 2015, a study used threshold-based discrimination for fNIRS signals and SVM-based classification for EEG signals to achieve 88% accurate results for self-paced MI tasks (Koo et al., 2015). In another work, a sensory motor rhythm-based paradigm was used to investigate the superiority of multi-modality for idle state detection (Lee et al., 2015). The LDA-based classification (achieving a 3.6% increase in the accuracy for MI tasks using the hybrid modality) showed that the NIRS signals contributed to the detection of the active/idle state as well as to the detection of active classes to confirm early activity detection. In two other similar studies, the MI of both the force and speed of hand clenching was decoded using hybrid EEG–NIRS (Yin et al., 2015b,c). In the first case, the extreme learning machine classifier was used to decode the responses associated with the force and speed imagery of the hand with an accuracy of 76.7%, whereas, for the second case (Yin et al., 2015c), the features of EEG and NIRS were combined and optimized using the joint mutual information selection criterion, again utilizing the extreme learning machines, in which case, the resulting average classification accuracy for the force and speed of hand clenching was 89%.

Several studies on the applications of hybrid EEG–NIRS have emerged in 2016, showing the trend of growing research in this area. The studies related to BCI applications were also discussed. In the case of active tasks, four motor tasks, namely right- and left-arm movement and right- and left-hand movement tasks, were decoded using the hybrid EEG–NIRS system (Buccino et al., 2016). Employing the LDA-based classification, the features using common spatial patterns (CSP) were compared with the signal mean (SM) and signal slope (SS). In the rest-task classification, the SM–SS average accuracy was 94.2% and the CSP average accuracy was 86.2%. The SM and SS, meanwhile, also performed better for right–left classification. SM–SS achieved an average accuracy of 72.2%, whereas with CSP, only 67.1% was possible. In the case of arm-hand classification though, CSP showed a better performance (83.6% average accuracy) than SM–SS (79.9%). In the case of passive tasks, the neural and hemodynamic correlates were estimated to measure drivers’ mental fatigue levels (Ahn et al., 2016). An average accuracy of 75.9% was achieved using an LDA-based classifier combining EEG, fNIRS, and ECG modalities. A new hybridization concept for combined EEG and fNIRS was introduced by Keles et al. (2016). In their study, different EEG bands (α, β, θ, and *Δ*) were estimated for the resting state. The correlation of an EEG band was convoluted with the modeled hemodynamic response (generated using two gamma functions) to generate the expected response with the incorporated neuronal activity for the hemodynamic signal. Since this was a pioneer study, its BCI-related role is yet to be investigated.

The most recent work on hybrid EEG–NIRS-based BCI demonstrates decoding of eight commands from the prefrontal and frontal cortices (Khan and Hong, 2017). In this work, four commands were decoded using EEG, in which two commands were generated using an eye blinking-based tasks and two commands were decoded using eye movement tasks. The interesting part of this study was the decoding of fNIRS commands in a 2 s window. For the selection of an optimal window size, the difference between the baseline and the first trial was used for channel selection. The signal mean and minimum values of HbO were used to detect the brain activity in that window. Four commands were generated using MA, mental counting, word formation, and mental rotation-based tasks, respectively. An average accuracy of 86% for EEG and that of 75.6% for fNIRS were achieved using an LDA-based classifier. This study was tested for the control of a drone, whose results showed the feasibility of using prefrontal-based commands for BCI.

## Combination of Brain Signals

The paradigm selection criterion for an hBCI system depends on the type of detected activities. As discussed earlier in the Section “Introduction,” BCI tasks are categorized into active, passive, and reactive types. The respective selection criteria for these tasks are based solely on the designed paradigm. In the case of multi-modality, the paradigm usually consists of the decoding of a single activity from the same brain region. Some hBCIs are designed by decoding multiple tasks using a single modality. For this purpose, usually SSVEP is combined with MI- or P300-based tasks using EEG-based signal detection. A study by Zhang et al. (2012) has demonstrated a combination of ERP-, N170-, and vertex-positive potential signals for EEG-based BCI. ERP-based tasks and evoked potential tasks are reactive, as they require external stimulation to generate the brain activity (Zander and Kothe, 2011). The MI- and MA-based tasks are considered active, as brain activity is generated by the user with internal brain activities. In this article, we briefly include the cases in which multiple tasks are detected simultaneously using a single modality, although the “hybrid” term is not used. This has been done in recent studies on fNIRS, wherein MI and MA tasks have been combined for the generation of multiple BCI commands (Hong et al., 2015; Naseer and Hong, 2015a). The various signals used in hBCI are discussed below.

### Signals Based on Audio and Visual Stimulation

These reactive signals are generated from either the occipital brain area or the temporal brain area by the provision of either visual stimuli (Liu and Hong, 2017) or auditory stimuli (Santosa et al., 2014; Hong and Santosa, 2016). Although mostly such stimulations are intended for the generation of brain activity from the corresponding lobes (An et al., 2014; Tidoni et al., 2014; Wu et al., 2016; Xu et al., 2016), some audio/video stimuli are given to generate P300 signals (Rutkowski, 2016). For healthy individuals, these stimulations can be effective in generating multiple commands. However, they can also be beneficial for patients with no motor or eye movements.

### SSVEP Signals

These signals are detected mostly in the occipital brain region. They are generated by gazing at a stimulus, which causes an increase in neural activity in the brain. VEPs are elicited by sudden visual stimuli, the repetition of which leads to a stable voltage oscillation pattern in EEG that is known as SSVEP. The stimulus used for these signals is light flickering at different frequencies (sometimes in the “checker board” pattern with changing colors). Using SSVEP signals, multiple reactive commands can be generated. The drawback of this activity is the need for the continuous focus on flashing light, which might not be possible or an ineffective approach for some patients (Muller-Putz et al., 2005). The signal detection time for these signals has been reduced to less than 1 s using spatio-temporal features with a reduced number of channels (Zhang et al., 2013, 2014; Chang et al., 2016; Wang et al., 2016).

### P300 Signals

This signal is detected mostly from the parietal brain region. They are the ERPs that indicate the responses to specific cognitive, or sensory, or motor events. The presentation of a stimulus in an oddball paradigm can produce a positive peak in EEG signals. This peak appears 300 ms after the onset of the stimulus. The stimulus can be visual, auditory, or somatosensory. This evoked response in EEG is the P300 component of ERP. These, most widely utilized in speller applications, also can generate multiple commands for BCI. However, being reactive, these signals are mostly used only for healthy subjects (Bayliss et al., 2004; Piccione et al., 2006; Turnip et al., 2011; Turnip and Hong, 2012).

### Prefrontal Signals

These signals are detected from the prefrontal and dorsolateral prefrontal brain regions. They are a good choice for BCI, as they require less training effort. In the case of fNIRS, they are especially suitable in that they incur fewer motion artifacts and less signal attenuation due to detector slippage in hair. Also, given their non-utilization of motor activities, they are more effective on patients with severe motor disabilities. MA, mental counting, and other tasks can be detected as active-type brain signals for BCI (Kim et al., 2012; Naseer et al., 2014, 2016a,b). The passive activity of drowsiness also can be detected from this cortex (Khan and Hong, 2015). Another research has reported the detection of music imagery, picture imagery, word generation, etc., from the prefrontal cortex (Naseer and Hong, 2015b; Ma et al., 2017). The most common task used for BCI purposes is MA.

### Motor Signals

These signals are detected mostly from the primary and central brain regions (mostly the motor cortex). They are most suitable for active BCI applications, as they are natural means of providing BCI control over external devices (Naseer and Hong, 2013). These signals are also targeted for the investigation of motor recovery or neuro-rehabilitation. They have wide applications in both EEG- and fNIRS-based BCI systems. Two different types of brain signals detected from the motor cortex are motor execution and MI. Motor execution is performed by the movement of muscles (mostly in hands or feet). Also, some eye-movement-related activities stimulate the motor region. Some BCIs use motor execution for the generation of commands, whereas in other cases, these signals are removed from EEG signals using EMG/EOG for better detection of MI. Motor imaging can be defined as a covert cognitive process of kinesthetic imagining of the movement of one’s own body without the involvement of muscular tension, contraction, or flexion (Naseer and Hong, 2015b). The MI signal is weak relative to the motor execution task. Also, not all subjects can perform this activity for BCI. In fact, since this activity is generated by the imagining of hand or foot movements, it is best suited for active-type BCI. A list of the different activities that can be used with a hybrid paradigm is provided in Table 2.

**Table 2:** Combinations of brain signals.

Task 1	Task 2	Sensor placement	Modalities	Activity type
Steady-state visual evoked potential (SSVEP)	P300	Occipital and parietal	Electroencephalography(EEG)	Reactive
SSVEP	Motor signals	Occipital and motor	EEG	Combination of reactive and active
Motor signals	P300	Parietal and motor	EEG	Combination of active and reactive
P300	Eye movement	Parietal, motor and eyes	EEG + electrooculography (EOG)	Reactive
Prefrontal signals	Motor signals	Prefrontal and motor	EEG + functional near infrared spectroscopy (fNIRS) (fNIRS individual in some cases)	Active for both individual fNIRS and fNIRS combined with EEG

### SSVEP and MI

The simultaneous decoding of SSVEP and MI signals constitutes a hybrid system consisting of active (based on MI) and reactive (based on SSVEP) commands. Although only a few relevant studies have appeared, they have successfully demonstrated the significance of the hybrid paradigm for control (Horki et al., 2011; Cao et al., 2014; Li et al., 2014) and rehabilitation applications (Daly et al., 2013; Yu et al., 2015).

#### Control Applications

The early work in this area demonstrated the control of a 2-DoF artificial limb using combined MI- and SSVEP-based tasks (Horki et al., 2011). The objective of this study was to operate the elbow and grasp movements of the artificial limb using BCI for spinal cord injury (SCI) patients. In this case, MI was used to control the grasp movements, whereas SSVEP was used to operate the elbow movements. According to their data, 87% of accuracy was achieved for the MI-based tasks and 91% of accuracy was achieved for SSVEP using LDA classification.

The control of a wheelchair using MI and SSVEP has been proposed in two studies (Cao et al., 2014; Li et al., 2014). In the first case (Cao et al., 2014), MI was used for the chair’s left and right turning and SSVEP was used for the speed control (three commands). An SVM-based classifier resulted in 90.6% of accurate control using the hybrid protocol. In the second case (Li et al., 2014), six commands were used to operate the wheelchair. Two commands were generated using MI (left and right rotations), and the remaining four commands (start, stop, forward, and backward movements) were generated using SSVEP.

In 2015, a study by Duan et al. (2015) demonstrated the control of a robot by combining SSVEP and MI tasks: SSVEP was used to generate three commands to make the robot move forward, turn left, and turn right, while MI was utilized to control the grasp motion of the robot. Meanwhile, CCA was used for SSVEP classification and power spectrum was employed to estimate the motor-imagery-related rhythms. The results suggested that the β-band was significant in MI. Overall, 80% accurate results were obtained for three subjects.

In terms of command generation, a study in 2016 proposed an improved tensor-based multi-class multi-modal scheme especially for EEG analysis in hybrid BCI (Ji et al., 2016). It combined SSVEP- and MI-based tasks for command generation. As per their method, the data need not be divided into individual groups and fed into separate processing procedures; rather, SVM was extended to multi-class classification for hybrid tasks. Applications in three datasets suggest that the proposed scheme not only can identify the different changes in the dynamics of brain oscillations induced by different types of tasks but also can capture the interactive effects of simultaneous tasks.

#### Motor Training

In the case of motor training, an initial investigation of hybrid MI-SSVEP was performed with cerebral palsy (CP) patients (Daly et al., 2013). The goal was to investigate the use of MI and SSVEP for CP. Six patients among 14 were able to exercise control *via* MI-based tasks, and three patients were able to exercise control *via* the SSVEP-based paradigm. The results served to demonstrate the potentiality of MI–SSVEP-based tasks for CP patients.

A recent study reported on MI training using SSVEP-based tasks with continuous feedback for an hBCI (Yu et al., 2015). During the initial training sessions, the subjects focused on the flickering buttons to evoke SSVEPs as they performed MI tasks. As the training progressed, the subjects were allowed to decrease their visual attention on the flickering buttons, provided that the feedback remained effective. The feedback was based mainly on motor imagery-based tasks. The results demonstrated that the subjects could generate distinguishable brain patterns of hand MI after only five training sessions lasting approximately 1.5 h each. An average accuracy of 89.03% was obtained after training using the hybrid paradigm with the LDA-based classifier.

### SSVEP and P300

The combination of SSVEP and P300 signals results in a reactive hBCI. These brain activities are simultaneously recorded from the occipital and parietal brain areas. This paradigm is used repeatedly in several control and rehabilitation applications.

#### Strategies for Signal Detection and Control

The initial step of hBCI can be established when multiple brain activities are simultaneously decoded for the detection of the patient’s intention (Panicker et al., 2011). In 2013, an hBCI was used for humanoid robot navigation using combined SSVEP and P300 signals (Choi and Jo, 2013). The data in this case were recorded from the motor, parietal, and visual cortices. The results of this experiment showed that a hybrid SSVEP–P300-based BCI can be used to navigate a robot with ease. In later works (Combaz and Van Hulle, 2015; Wang et al., 2015), simultaneous detection of SSVEP–P300 has been reported for oddball as well as shape- and color-changing paradigms. In 2014, the hybrid SSVEP–P300-based paradigm was used in the development of speed- and direction-based cursor control (Bi et al., 2014). In this case, the stimuli for P300 were distributed at the top and bottom edges of the screen, whereas the stimuli (accessed by turning control knobs clockwise or counter-clockwise) for detection of the SSVEP signals were shown on the left and right sides of the screen. Their SVM-based classification showed an accuracy of above 90% for hBCI.

Awareness of the patients with consciousness disorders has also been detected using the combined paradigm of SSVEP and P300 (Pan et al., 2014). In this case, two photos were presented to each patient: one was the patient’s own photo and the other was unfamiliar ones. The patients were instructed to focus either on their own or on the unfamiliar photo. The BCI system determined which photo the patient focused on by using both P300 and SSVEP features. Eight patients [four in a vegetative state (VS), three in a minimally conscious state (MCS), and one in a locked-in syndrome (LIS) state] participated in the experiment. Using SVM-based classification, one of the four VS patients, one of the three MCS patients, and the LIS patient were able to selectively attend to their own or unfamiliar photos (classification accuracy, 66–100%). Two additional patients (one VS and one MCS) failed to attend to unfamiliar photos (50–52%) but achieved significant accuracies for their own photos (64–68%). Finally, the other three patients failed to show any significant response to the commands (46–55%). These results strongly support the necessity to use an hBCI paradigm for patients. In another study (Allison et al., 2014), a four-choice selection scheme was developed resulting in an improved accuracy using P300 and SSVEP. In order to generate P300 signals, the subjects had to focus on one of the four boxes that were displayed on the screen. The shown boxes change their color from red to white (one box at a time) for 100 ms with a 25 ms delay for the next flash. A 4 s trial was used to record P300 signals. To generate SSVEP, the boxes were flickered instead of being flashed for 4 s. The flickering frequency was 6, 8, 9, and 10 Hz. The subjects were asked to focus on one box and count the number of flickers to simultaneously generate the hybrid signals. LDA was used for P300 signal classification, while CCA was used to classify SSVEP signals. When classified separately in their hybrid paradigm, the average accuracy for p300 was about 99.9%; however, the accuracy for SSVEP was 67.2%.

The importance of the reactive task has been shown in a comparative study of telepresence-robot and humanoid robot control (Zhao et al., 2015). Four-class SSVEP and six-class P300 achieved an average accuracy of 90.3 and 91.3%, respectively, using LDA as a classifier. For wheelchair control, the hybrid SSVEP–P300 study (Li et al., 2013) used buttons flickering in a graphical user interface (GUI). The movement options were selected by focusing on the selected direction of the GUI. The SVM-based classification resulted in high accuracy (> 80%), thus demonstrating the importance of the hBCI paradigm for wheelchair control. In another work (Wang et al., 2014), blink signals (EOG) were also added as a part of hBCI for wheelchair control.

Most recently, a new scheme that uses the steady-state somatosensory evoked potentials (SSSEPs) has emerged (Breitwieser et al., 2016; Pokorny et al., 2016). The hBCI in these cases combines SSSEPs and P300 potentials evoked by twitches randomly embedded into the streams of tactile stimuli. The twitches are given in the form of stimulation to the left-/right-hand index finger. Both of the mentioned studies have used LDA for classification. Pokorny et al. (2016) showed that the accuracies of SSSEP and P300 were 48.6 and 50.7%, respectively. However, combining the features of SSSEP and P300 resulted in 55.5% average accuracy for the twitching task. Hybridization related to SSSEP-based tasks is relatively new, and its full potential is yet to be determined.

#### Hybrid Strategies for Spellers

A speller paradigm is based on a combination of rows and columns displayed and flickered for command generation. The first study on a hybrid SSVEP–P300-based speller paradigm (Xu et al., 2013) used the combination of SSVEP and P300 features. Its aim was to distinguish the SSVEP and P300 activities in the brain by monitoring the data from the motor, parietal, and occipital brain regions. The results showed that during a non-target phase, SSVEP activity was evident, but after the target stimuli were given, it was replaced by P300 potentials. SSVEP-B (sub-signals in the absence of SSVEP) mostly appears in the occipital region (Oz), which can be compared with P300 activity in the motor region (Cz). Another work (Yin et al., 2013) employed random flashing and periodic flickering to evoke P300 and SSVEP simultaneously. This was done to increase the differences between the row and column symbols. A SW-LDA was used to achieve an average accuracy of 93.85%. Another study of using such SW-LDA (Xu et al., 2014) achieved an average accuracy of 96.8% for P300 and 95.7% for SSVEP. In this case, the SSVEP–P300 activities were decoded in parallel. Four flashing patterns were used to detect the SSVEP, and the speller characters were divided into four blocks. A block was selected using SSVEP, and the characters were selected using P300.

A high accuracy (>90%) was obtained using SW-LDA in a speedy hBCI spelling approach (Yin et al., 2014). To evoke the P300 and SSVEP potentials simultaneously, this study used flash-pattern mechanisms composed of random flashings and periodic flickering. The random flashings were created by highlighting items using orange crosses in a pseudorandom sequence, and the periodic flickering was achieved using white rectangular objects alternately appearing on and disappearing from a black background. The use of the speller-based paradigm in the form of a hybrid SSVEP/P300 system for the selection of 64 options (64 control-command generations) in BCI has been reported (Yin et al., 2015a). In this study, for SSVEP classification and SW-LDA for P300 signals, the canonical cross-correlation approach was used. Also, maximum probability estimation was utilized for data fusion, which resulted in a 95.18% average accuracy.

In an attempt to navigate a vehicle to its destination, the use of the SSVEP–P300-based speller paradigm above has been reported (Fan et al., 2015). Specifically, the speller was used for entering the destination location and flickering “checker board” stimuli (12 Hz and 13 Hz) were used for destination selection and deselection, in which LDA-based classification achieved an average accuracy of 98.09% for real-driving conditions.

### MI and P300

The MI- and P300-related tasks have been widely designed for applications in real-world environment (Li et al., 2010; Su et al., 2011; Long et al., 2012a,b; Yu et al., 2012, 2013; Bhattacharyya et al., 2014; Naito et al., 2014; Kee et al., 2015; Zhang et al., 2016a). The corresponding signals are obtained by positioning electrodes around the motor and parietal brain regions. In the work of Li et al. (2010), MI and P300 were combined for the control of a cursor on the screen using SVM as a classifier (90.75% average accuracy). In the work of Long et al. (2012b), target selection or rejection (mouse-clicking) features were added resulting in the average accuracy of 92.8%.

Navigation and target selection in a virtual environment was demonstrated in Su et al. (2011) using the hybrid MI–P300-based paradigm. In their work, MI was used for navigation and P300 for target selection (three control buttons). Overall, five commands were generated using Fisher LDA- and SVM-based classification (84.5% for MI and 81.7% for P300).

In the work of Long et al. (2012a), the same hybrid paradigm was used to control the direction and the speed of a simulated and afterward a real wheelchair. The turning (left and right directions) controls were associated with left- and right-hand imageries. The wheelchair was decelerated using a foot-movement imagery. The acceleration was represented using P300 signals. In this case, the LDA-based classification resulted in 71.6% accuracy for MI and 80.4% for P300. Another study of Yu et al. (2012) demonstrated the utility of hybrid MI–P300 for cursor-controlled on-screen feature selection-based Internet surfing. In that study, SVM-based classification resulted in an average accuracy of 93.2%.

In 2013, a real-time electronic mail communication system was implemented to enable clients/users to receive, write, and attach files to their email (Yu et al., 2013). According to their hybrid MI–P300 paradigm, the SVM-based classifier yielded a high accuracy (average: >90%) for the system.

In 2014, robot arm movement control for rehabilitation was implemented using the hybrid method (Bhattacharyya et al., 2014). Arm movement was controlled by MI signals, while P300 was used to detect the stopping intention. A 95% success rate was achieved in the SVM-based classification. The recent work on this paradigm has focused on optimal feature selection and simultaneous classification methods (Naito et al., 2014; Kee et al., 2015). Also, the use of optimal feature selection for the enhancement of output accuracy was another issue in Naito et al. (2014).

In 2015, GA-based strategy was proposed for the optimization of channel selection in the process of simultaneous recording of MI and P300 (Kee et al., 2015). A recent contribution of 2016 proposed an autonomous wheelchair navigation system that acquires the destination and waypoint based on the existing environment (Zhang et al., 2016a). In this case, the BCI module selects and determines the destination using MI and P300, respectively.

### Mental and Motor Tasks

For mental VS motor task purposes, paradigms are designed to obtain the brain activities from the prefrontal and motor cortices. Both EEG and fNIRS have provided good results for these tasks. In these cases, mostly a working memory-related task is combined with a motor task to achieve the BCI system. For the localization of neuronal sources, EEG was applied using six cognitive tasks (arithmetic, navigation imagery, auditory recall, phone imagery, and MI of the left and right hands) and compared against the idle state to localize the brain location (Dyson et al., 2010). The spatial areas suggested a clear discrimination between the arithmetic- and auditory-related tasks, while the MI-related tasks were discriminated according to the baseline.

As for fNIRS, MA has been combined with music imagery for simultaneous decoding of brain activity (Power et al., 2010, 2011; Stangl et al., 2013). In this case, however, the prefrontal channels were averaged and the activities were differentiated using an HMM-based classification method. MA and MI have also been reported to be combined in the case of fNIRS. Four commands have been generated by simultaneous decoding of mental counting-, arithmetic-, and imagery-related tasks (Hong et al., 2015; Naseer and Hong, 2015a). In these cases, LDA-based classification was used to decode the activities from the prefrontal and motor cortices.

In a multi-modality case, meanwhile, four commands were generated by decoding mental tasks (MA and mental counting) using fNIRS and motor tasks (left- and right-hand tapping) using EEG for hBCI (Khan et al., 2014). This work was later extended for the decoding of eight commands using eye movement and mental tasks (Khan and Hong, 2017). In this case, EEG was employed to decode two and three eye blinks and left- and right-eye movements, whereas fNIRS was used to decode mental arithmetic-, mental counting-, word formation-, and mental rotation-based tasks. The decoded eight commands were used to operate a quadcopter in the 3D space.

### Hybrid Audio–Visual Tasks

In this category, there is not much hybrid research. In one work (Putze et al., 2014), hybrid EEG–NIRS was used to discriminate the auditory and visual stimuli. The details on this study can be found in the above “EEG + NIRS” section. Tidoni et al. (2014) used audio feedback to improve the performance of the BCI system. Six commands were generated using SSVEP-based tasks for a robot’s pick-up and placing tasks. It is found that audio–visual synchrony between footstep sounds and actual humanoid walking reduces the time required for steering the robot. This demonstrates the possibility of using auditory feedback congruent with humanoid actions to improve the motor decisions of the BCI. An et al. (2014) used six auditory stimuli for the selection of 36 visual symbols using EEG recording for a gaze-independent BCI. When a subject focuses on a sound, a selection screen consisting of six symbols is shown. The subject can then choose one from the six visual symbols for choice selection. This paradigm increased mental fatigue, as the user has to focus on the audio cues. An LDA-based scheme was used, and 87.7% of accurate results were generated. In another work of Barbosa et al. (2016), in contrast to An et al. (2014), a P300-based BCI was used for combining visual and audio stimuli. In this case, the audio stimuli were natural spoken words, which reduced the mental work load. The average online accuracy for their hybrid approach was 85.3%, which represents an improvement of 32% relative to independent audio or visual stimuli.

## Advantages of hBCI

Although the combination of two modalities increases the system cost, its efficiency is significantly improved. Since most BCI systems are designed for the purpose of rehabilitation or commutation (of patients), hBCI is a better mean in achieving this goal. A complete BCI that can be used by patients is yet to be designed. However, the combination of the modalities can provide the first step toward the goal. Each hBCI has different advantages and applications. The use of EEG–EOG and EEG–EMG systems, for example, are viable for patients capable of minor eye and muscular movements, whereas a different approach is required for completely locked-in patients. In this regard, hybrid EEG–fNIRS might provide better results. In other words, whereas the advantages of hBCI vary with the combination of modalities, the main goal remains the same.

### Minimization of False Signal Detection

The BCI research has demonstrated the use of EEG in several communication and control applications. In most cases, MI was used to generate the commands for communication (Machado et al., 2010; Bi et al., 2013; Hwang et al., 2013; Ahn and Jun, 2015; Maria Alonso-Valerdi et al., 2015). However, it is difficult for patients to perform an MI activity. Also, the detection of MI signals requires extensive processing, and false detection can result in severe consequences in real environments. Long-term use of SSVEP and P300 can also increase visual fatigue of the subjects, thereby incurring a false detection of signals for BCI. Thus, proper measures are required to increase the accuracy of the system by minimizing the false detection rate. This can be achieved by combining multiple modalities. In such a case, simultaneous feature decoding results in a better system accuracy. The most common example is the hybrid EEG–fNIRS.

### Greater Suitability for BCI

The number of active commands used for system control is a central issue for BCIs. The main problem in EEG-based BCI systems is the loss of accuracy resulting from an increased number of (active) commands. Although the number of commands can be increased using reactive tasks, it is difficult to make the patient concentrate on reactive tasks for a long duration. Although strategies are being designed to overcome this problem, the results have not yet been proved sufficiently effective for BCI adoption (Lesenfants et al., 2014). In this context, the hBCI plays an important role in providing the potential for an increased number of commands without undo-influence on classification accuracy. The approach most widely employed in the brain signal-based control of wheelchairs is to increase the number of commands by decoding the features from two modalities separately.

Tables 3–5 list all of the important studies from 2010 to 2016 that have combined two modalities to decrease false detection and enhance classification accuracy for BCI. The tables show the relevant hBCI studies for the enhancement of accuracy and increase in control commands. Ideas on increasing accuracy and the total number of commands can be deduced from them. We divided the tables into active, passive, and reactive BCI categories. This information can be helpful to the selection of brain signal acquisition modalities based on the types of activities. Also, we have incorporated the classifier and window size information in each table, which might be helpful to prospective researchers looking a method to enhance classification accuracy and increase the number of commands.

**Table 3:** Important active hybrid brain–computer interface studies with applications to increased accuracy and number of commands for brain–computer interface studies (BCI) (from 2010 to 2016).

Reference	Brain area	Activity	Modality	Application	Analysis type	Classifier	Commands	Accuracy	Window size
Li et al. (2010)	Whole brain	Motor imagery (MI) and P300	Electroencephalography (EEG) + electrooculography (EOG)	Cursor control in 2D	Online	Support vector machine (SVM)	4	92.8%	0–600 ms after button flashes on the screen for 8 s

Allison et al. (2010)	Motor and occipital regions	MI and steady-state visual evoked potential (SSVEP)	EEG	Option selection from the screen	Offline	Linear discriminant analysis (LDA)	4	74.8% for MI, 76.9% for SSVEP, and 81% for hybrid	3–5 s window

Zhang et al. (2010)	Motor, parietal, and occipital regions	Mental task	EEG + EOG + electromyography (EMG)	Application to devices control	Offline	Fisher discriminant analysis combined with Mahalanobis distance	4	75.3% average for two-class and 54.1% for four-class	0–1 s

Su et al. (2011)	Whole brain	MI and P300	EEG	Virtual environment control	Online	SVM and fisher LDA	5	84.5% for MI and 81.7% for P300	0–2 s for MI and 0.7 s for P300

Leeb et al. (2011)	Motor cortex	Motor execution	EEG + EMG	Application to patient motor training	Online	Bayesian	2	87% for individual and 91% for hybrid case	0.5 s for EEG and 0.3 s for EMG

Long et al. (2012a)	Frontal, central, parietal, and occipital regions	P300 and MI	EEG	Direction and speed control for wheelchair	Online	LDA	5	75.4% for hybrid task	1 s

Yong et al. (2012)	Motor cortex	Hand and eye movement	EEG + EOG (eye tracker)	Artifact removal for choice selection	Online	SW-LDA	2	True positive rate increases from 44.7 to 73.1% (in 1 s)	1 s

Fazli et al. (2012)	Frontal, motor, and parietal cortex	MI and Motor execution	EEG + functional near infrared spectroscopy (fNIRS)	Application to control	Offline	LDA	2	93.2% (motor execution) and 83.2% (MI)	0.75 s for EEG, 6 s prior to stimulus onset and up to 15 s after stimulus onset using 1 s sliding window for fNIRS

Choi and Jo (2013)	Whole brain	SSVEP, MI, and P300	EEG	Humanoid robot navigation and recognition	Real time	CCA	6	84.6% for P300 and 84.04% for SSVEP	2 s

Cao et al. (2014)	Frontal, central, parietal and occipital cortex	SSVEP and MI	EEG	Brain-actuated switch for wheelchair control	Online	SVM	8	90.6%	–

Wang et al. (2014)	Whole brain	MI, P300 and eye blinking	EEG + EOG	Asynchronous wheelchair control	Online	SVM	7	91, 93, 89, and 92% for forward, backward, stop with special threshold, and stop with optimal threshold, respectively	4 s

Khan et al. (2014)	Prefrontal and motor cortex	Mental arithmetic, mental counting and motor execution	EEG + fNIRS	Application to wheelchair control	Online	LDA	4	94.7% for left and right movement commands (EEG) and 80.2 and 83.6% for forward and backward using fNIRS	0–10 s for fNIRS and 0–1 s for EEG

Kim et al. (2014)	Complete brain	Eye movement	EEG + Eye tracker	Quadcopter control	Real time	SVM	8	91.67%	5 s

Jiang et al. (2014)	Motor cortex	MI and eye movement	EEG + EOG	Application to BCI control	Online	LDA	4	90.4% for MI, 91.1% for relax, 96.4% for gaze left, and 97.3% for gaze right	3 s

Kaiser et al. (2014)	Motor cortex	MI	EEG + fNIRS	Application to brain monitoring	Online	LDA	1	3.6% increase in accuracy by hybrid modality	3–7 s

Lorenz et al. (2014)	Whole brain	ERP and MI	EEG	BCI driven neuro-prosthesis	Online	LDA	6	Maximum selection accuracy of 98.46% and maximum confirmation accuracy of 96.26%	1 s

Blokland et al. (2014)	Motor cortex	MI and motor execution	EEG + fNIRS	Application to tetraplegia patients	Offline	–	2	87% for motor attempt and 79% for MI in tetraplegia patients	3–15 s for fNIRS and 0–15 s for EEG

Bai et al. (2015)	Whole brain	MI and P300	EEG	Opening, closing, selection of files on explorer	Online	SVM	9 (can achieve 50)	>90%	4 s window for MI and 600 m for P300

Hortal et al. (2015)	Motor and parietal cortex	Mental imagination	EEG + EOG	Robotic arm control for pick and place task	Real time	SVM	6	Task 1: 71.13% and Task 2: 61.51%	0.5 s to synchronize output to BMI

Hong et al. (2015)	Prefrontal and motor cortex	Mental arithmetic and MI	fNIRS	Applications to three choice selection	Offline	LDA	3	75.6%	2–7 s

Naseer and Hong (2015a)	Prefrontal and motor cortex	Mental arithmetic, mental counting and MI	fNIRS	Decoding answers to four-choice questions	Offline	LDA	4	RMI, LMI, MA, and MC were correctly classified as 72.9, 64.2, 65.1, and 71.0%, respectively	2–7 s

Yin et al. (2015c)	Motor cortex	MI task	EEG + fNIRS	Increase in accuracy for BCI	Online	ELM	2	88%	0.5 s for EEG and 0–12 s for fNIRS

Koo et al. (2015)	Motor cortex	Self-paced MI	EEG + fNIRS	Application to device control	Online	SVM	2	88% average accuracy	10 s for fNIRS and three 5 s time windows with step size of 2.5 s for EEG

Buccino et al. (2016)	Motor cortex	Arm and hand movement	EEG + fNIRS	Hand movement discrimination	Online	LDA	2 commands simultaneously	94.2% (for rest-task classification)	0~6 s hybrid

Shishkin et al. (2016)	Whole brain	Eye gaze	EEG + EOG	Game control	Offline	LDA	–	90%	0.3 s for EEG and 0.2–0.5 s for EOG

Khan and Hong (2017)	Frontal	Mental task and eye movement	NIRS + EEG	Applications to quadcopter control	Online	LDA	8	76.5% for NIRS and 86% for EEG	1 s for EEG and 2 s for NIRS

**Table 4:** Important reactive hybrid brain–computer interface studies (from 2010 to 2016).

Reference	Brain area	Activity	Modality	Application	Analysis type	Classifier	Commands	Accuracy	Window size
Yin et al. (2013)	Parietal and occipital cortex	P300 and steady-state visual evoked potential (SSVEP)	Electroencephalography (EEG)	Speller	Online	SW-LDA	Up to 36	93.85% using hybrid paradigm	All rows and columns were flashed in 2.88 s

Zimmermann et al. (2013)	Motor cortex	Isometric finger-pinching task	fNIRS + bio-signals (ECG)	Feasibility for BCI	Offline	Hidden Markov model (HMM)	1	88.5%	5–20 s

Li et al. (2013)	Whole brain	SSVEP and P300	EEG	Wheelchair control	Online	Support vector machine (SVM)	6	>80%	0–0.6 s after a button flash complete for P300 and 3.2 s for SSVEP

Xu et al. (2013)	Whole brain	SSVEP and P300	EEG	BCI speller for target selection	Online	SW-LDA	9	93.3% for P300 + SSVEP-B	0–0.8 s after the onset

Bi et al. (2014)	Parietal and occipital cortex	P300 and SSVEP	EEG	Speed and direction for cursor control	Online	SVM	4	>90	4 s

Aziz et al. (2014)	Frontal and occipital	Eye movements	EEG + electrooculography (EOG)	Automated wheelchair navigation	Online	SVM, HMM	5	98%	0.5 s

Li et al. (2014)	Motor and occipital	Motor imagery and SSVEP	EEG	Wheelchair control	Real time	SVM	6	−	−

Witkowski et al. (2014)	Motor cortex	Hand-grasping motion assisted with exoskeleton	EEG + EOG	Assistive rehabilitation applications	Online	Sensitivity index	4	Average accuracy 62.28% for two conditions	5 s

Putze et al. (2014)	Auditory and visual cortex	Visual and auditory stimuli	EEG + functional near infrared spectroscopy (fNIRS)	Application to patient choice selection	Online	Linear discriminant analysis (LDA), SVM	2	94.7% average	Four window sizes 1, 2, 4, 8, and 16 s

Tomita et al. (2014)	Visual cortex	SSVEP-based task	EEG + fNIRS	Optimal window selection for hybrid EEG–NIRS	Offline	−	1	85% average accuracy (in 10 sec optimal window)	0–10 s

Fan et al. (2015)	Parietal and occipital	SSVEP and P300	EEG	Vehicle destination selection system	Online	LDA	11	99%	0–0.51 sec from onset for P300 and 8 s for SSVEP

Ma et al. (2015)	Parietal and occipital	P300 and eye blink	EEG + EOG	Mobile robot control	Real time	LDA	9	87.3% for average of five trials	~1.6 s

Combaz and Van Hulle (2015)	Whole brain	P300 and SSVEP	EEG	Applications to locked-in patients option selection	Online	SVM	12	Maximum achieved > 95%	200 ms before stimulation to 800 ms after stimulation for experiment 1

Wang et al. (2015)	Whole brain	P300 and SSVEP (shape changing and flickering-hybrid)	EEG	Development of new paradigm with application to devices control	Online	canonical correlation analysis (CCA), Bayesian LDA	4	Overall 20% increase in SSVEP classification, 100% for P300	Flash start to the flash end for SSVEP, single flashes lasting 0.8 s for P300

Ramli et al. (2015)	Motor and occipital	Eye gaze	EEG + EOG	Application to BCI applications (wheelchair control)	Online	Finite-state machine (FSM)	6	97.88%	0.5 s

Yin et al. (2015a)	Parietal and occipital cortex	P300 and SSVEP	EEG	Speller paradigm with applications to BCI systems control	Online	SW-LDA for P300, CCA for SSVEP	Up to 64	95.18%	0.8 s epochs after stimulation

Kim et al. (2016)	Occipital	SSVEP and eye movement	EEG + EOG	Turtle movement control	Online	CCA	4	83% for event-related desynchronization (ERD) and 92.7% for SSVEP	2 s

Lin et al. (2016)	Occipital	SSVEP	EEG + EMG	Choice selection	Online	CCA	2	81%	0.5–5 s

**Table 5:** Important passive hybrid brain–computer interface studies for drowsiness detection (from 2010 to 2016).

Reference	Brain area	Modality	Application	Analysis type	Classifier	Commands	Accuracy (%)	Window size (s)
Khushaba et al. (2011)	Frontal and occipital	Electroencephalography (EEG) + electrooculography (EOG) + ECG	Driver drowsiness detection	Online	Linear discriminant analysis (LDA), support vector machine (SVM), K-nearest neighbor, and kernel SVM	1	95−97	10
Chen et al. (2015)	Frontal and occipital	EEG + EOG	Automatic detection of drowsiness	Online	ELM	2 (single command for drowsiness)	97.3	8
Ahn et al. (2016)	Whole brain	EEG + NIRS	Mental fatigue level estimation	Online	LDA	1	75.9	60

## Applications

In recent years, significant progress has been made in hBCI research. Although some studies have demonstrated a success in wheelchair (and other devices) controls, most of them have involved healthy subjects. The true potentials of those modes of control, then, cannot be considered to have been fully discovered. An hBCI increases the classification accuracy (e.g., EEG + fNIRS), but this results in slower command generation. In cases where the number of commands is increased (e.g., EEG + EOG), a uniform window for command generation is needed. These are additional drawbacks of hBCI that have yet to be addressed. Also, most hBCIs were tested in a controlled laboratory environment where the user can comfortably concentrate on mental tasks, whereas in real situations, a high performance of concentration-dependent mental tasks (e.g., MI and MA) is much more challenging.

### Hybrid BCI for Patients

The ultimate goal of a BCI system is to provide assistance to patients (Kim et al., 2011). This assistance can be in the form of formulating a methodology that can be used to communicate with the environment. The patient should be able to express his/her thoughts through the use of the BCI system. Regarding rehabilitation, a BCI system has the capacity to distinguish improvement from non-improvement as a result of therapy and brain stimulation. Detection of seizures (Nguyen et al., 2012, 2013), epilepsy (Peng et al., 2014; Pouliot et al., 2014; Visani et al., 2015), and estimation of improvement in motor functions after stroke (Das et al., 2016) are such examples pursuing hBCI for patients. The current BCI system, however, lacks the potential to provide detailed functions. In fact, an hBCI can be a more powerful tool than a single-modality BCI, as it can provide more reliable information for control and rehabilitation applications for patients. Indeed, the recent hybrid combinations have shown successes. The hBCI can be more successful for patients in the following areas.

#### Neuro-Rehabilitation

Hybrid brain–computer interface systems can be used to restore some of the lost motor and/or cognitive functions for individuals with stroke and SCI. Neuro-feedback is required to train individuals to self-regulate the brain activity (Weyand et al., 2015). Although fNIRS has demonstrated the effectiveness for brain activity monitoring (Kassab et al., 2015), the use of both EEG (Gruzelier, 2014) and fMRI (Laconte, 2011; Weiskopf, 2012) has been more widely reported. EEG is employed due its high temporal resolution, whereas fMRI is preferred due to its high spatial resolution. However, EEG suffers from the limitations of imprecise localization and the inaccessibility of subcortical areas, while fMRI is slower in the detection of hemodynamic activity. Hybrid EEG–NIRS-based hBCI, then, is most suited for these cases. Indeed, the spatial and temporal issues are resolved by the combination of the two modalities. Also, it provides the advantage of simultaneous monitoring of electrophysiological and hemodynamic signal monitoring. For neuro-rehabilitation purposes moreover, hybrid EEG–NIRS’s use of neuro-feedback in MI-based tasks has been successfully demonstrated (Kaiser et al., 2014). Most recently, fNIRS–EEG study has shown its contribution for refractory epilepsy patients (Vannasing et al., 2016). This case study demonstrated the potential of NIRS to contribute favorably to the localization of language functions in children with epilepsy and cognitive or behavioral problems and showed, moreover, its potential advantages over fMRI in pre-surgical assessment.

#### Communication and Control

The major role of BCI is to serve as a mean of communication for patients with motor disorders (e.g., LIS and SCI). For this purpose, different approaches have been demonstrated using hBCI. FES and EEG have been combined with brain signal acquisition systems for motor restoration (Rohm et al., 2013). In this case, the patients were trained using FES- and MI-based tasks. One year of training resulted in 70.5% accuracy of MI tasks for SCI patients. Lim et al. (2013) developed a system that allows users to express their binary intention without need to open their eyes. A pair of glasses with two light emitting diodes flickering at different frequencies was used to present visual stimuli to participants with their eyes closed. The binary commands were generated using SSVEP. This system showed 80% accurate results for ALS patients. An alternative use of EEG–fNIRS as a brain switch has also been reported for tetraplegia patients (see the “EEG + NIRS” section for details). Blokland et al. (2014) decoded two “yes/no” responses from tetraplegia patients. Although the command generation time was slow, this study showed the significance of using hybrid EEG–NIRS for patients. Hybrid SSVEP–P300-based paradigms for the investigation of consciousness disorder in patients have been reported (see the “SSVEP and P300” section for details). Also, gaze-independent hBCI using visual and auditory stimuli with P300-based tasks has been proposed for LIS patients (Barbosa et al., 2016).

#### Motor Therapy and Recovery

The monitoring of the brain state during brain stimulation is an important application of hBCI systems. Transcranial magnetic stimulation and transcranial direct current stimulation (tDCS) are used to stimulate the brain for therapy. The electrical brain activity for motor recovery estimation was monitored using EEG (Zaghi et al., 2010; Schestatsky et al., 2013; Sale et al., 2015). Also, the hemodynamic response was monitored using fNIRS (Faress and Chau, 2013; Khan et al., 2013; Ishikuro et al., 2014). Combined EEG–fNIRS provides an edge over the individual modalities in that electrical and hemodynamic responses can be monitored simultaneously (Dutta, 2015; Dutta et al., 2015; Jindal et al., 2015). This application, certainly, can facilitate medical diagnoses for the purposes of motor therapy and recovery. A lot of research has been carried out in this area for stroke patients. The most recent work in this context has used EEG–NIRS joint imaging for the measurement of the brain recovery of stroke patients after tDCS stimulation (Das et al., 2016; Guhathakurta and Dutta, 2016; Sood et al., 2016).

#### Infants Brain Monitoring

The monitoring of brain development is essential for infants. It helps in avoiding several brain disorders in developing children. Although, a single brain imaging technique may help to monitor autism spectrum disorder, attention-deficit hyper-activity disorder, and speech and language impairments (Aslin et al., 2015; Sperdin and Schaer, 2016), hybrid systems may provide a better diagnosis for such disorders. Also, the brain development of neonates can be better understood by simultaneously measuring neuronal and hemodynamic brain activities.

### Hybrid BCI for Healthy Individuals

As per the above discussion, it seems that hBCI/BCI is more suited to patients; however, most studies have used healthy subjects for experimentation in a lab environment. This might be due to the fact that hBCI is still in its developmental phase. However, in our opinion, hBCI has several aspects that are most suited to healthy people. The following are the three major applications of hBCI.

#### Control Applications

The hBCI can be useful in environment control settings for healthy individuals. Environment control is very helpful for those who need to do multiple tasks utilizing several devices (e.g., remote control and light control). Using brain signals, a person can perform these tasks remotely, for which operations, high accuracy is required; thus, in such scenarios, hBCIs can be effective. Also, using hBCI schemes, a robot can be controlled remotely to perform several tasks. For amputees, an hBCI, relative to a single modality, can be a more effective and reliable communication tool for the control of prosthetic devices, as it can achieve higher accuracy. For example, Hwang et al. (2012) developed a mental spelling system based on SSVEP, adopting a QWERTY style layout keyboard with 30 LEDs flickering with different frequencies. The mental spelling system allows the users to spell one target character per each target selection. A total of 87.58% accurate results were achieved by their study.

#### Entertainment

Recently, BCIs also have been employed for healthy individuals’ entertainment purposes (Ahn et al., 2014; Bai et al., 2015; Li et al., 2016), though this is not the main priority of BCI research. In any case, the feasibility of brain-controlled video games has been demonstrated using EEG-BCI; however, no actual hBCI application has been introduced to date yet. In any event, it should be emphasized that for training purposes, such games might be useful in generating desired brain activities that can be decoded using hBCI modalities.

#### Safety

Perhaps hBCI’s main application is safety. Indeed, first and foremost, it can be useful in monitoring the vigilance levels of pilots and drivers. For a pilot confronting an emergency landing, the monitoring of the exact mental status of the pilot can contribute to a safe landing. Although commercial systems that can monitor brain activity and alert drowsy drivers do not yet exist, hBCI might nonetheless contribute to the development of such a commercial system. In the case of tele-operated robots, hBCI can be very effective in monitoring the anxiety levels of doctors. This could be a useful approach, especially for complicated surgeries.

#### Neuro-ergonomics

Neuro-ergonomics is the study of human brain in relation to performance at work and everyday setting. EEG is most widely used in measuring the passive brain states (Qian et al., 2016, 2017). Most recently, fNIRS has also proven to be a viable candidate for passive brain activities detection (Ayaz et al., 2012, 2013; Khan and Hong, 2015). Hybrid BCI systems may give better information about the physical fatigue, cognitive functions, mental workload, vigilance, and mental fatigue of a person. This can be helpful to the person to avoid extreme workloads and loss of vigilance.

## Future Perspectives

**Figure 6:**
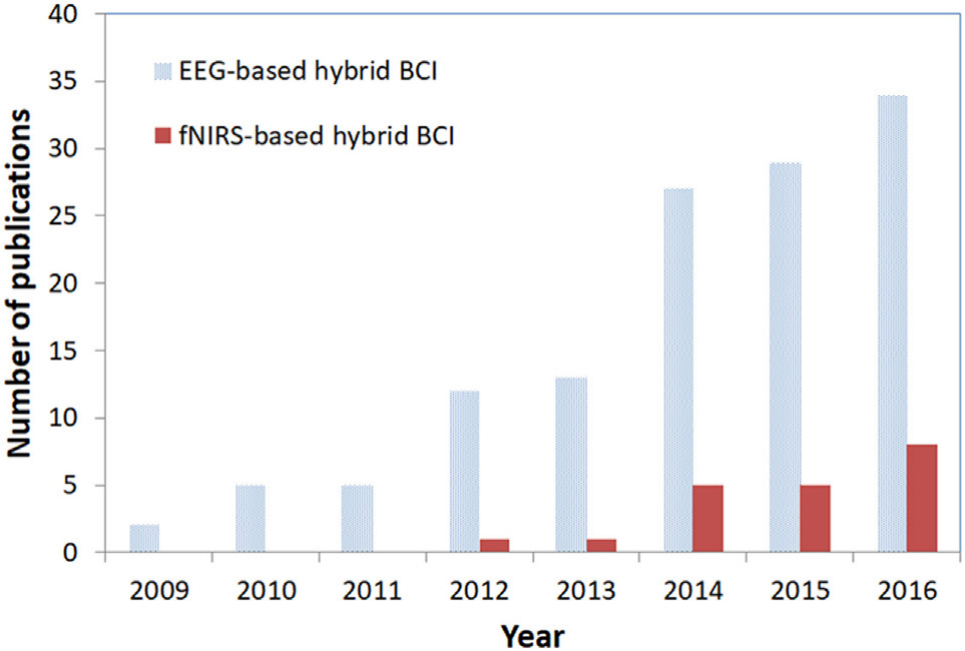
Trend in electroencephalography (EEG)/functional near infrared spectroscopy (fNIRS)-based hybrid brain–computer interface (BCI).

The research on hBCI has begun to increase in recent years. Although the hBCI scheme emerged before 2010, a major acceleration in the derivation of developmental strategies has been observed only in the previous 2 years. Most hybridization strategies that have been introduced are applicable to EEG-based BCI; yet, further improvement of fNIRS-based BCI systems is needed. Figure 6 shows the recent trend in EEG- and fNIRS-based hBCIs.

**Figure 7:**
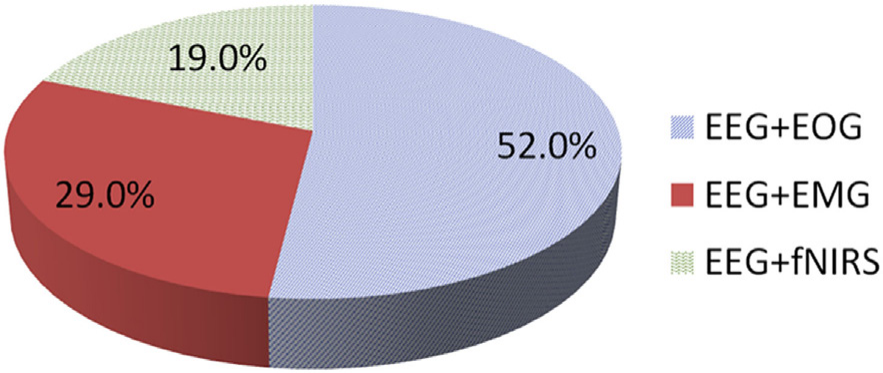
Hybrid brain–computer interface using electroencephalography (EEG) in combination with other modalities (2009–2016).

The major hBCI emphasis is the EEG–EOG-based hBCI. Most of these studies have combined, or are combining, two modalities for eye movement artifact removal and additional BCI commands. EEG–EMG-based hBCIs have limited applications and are used only in muscular-artifact removal from brain data for enhanced classification accuracy. Meanwhile, only very limited research has been done on EEG–fNIRS-based BCI applications. Moreover, the works done have focused mostly on an improvement of classification accuracy, with very little attention having been paid to the issue of command-number increase. A breakdown of the hBCI application approaches introduced from 2009 to 2016 is provided in Figure 7.

**Figure 8:**
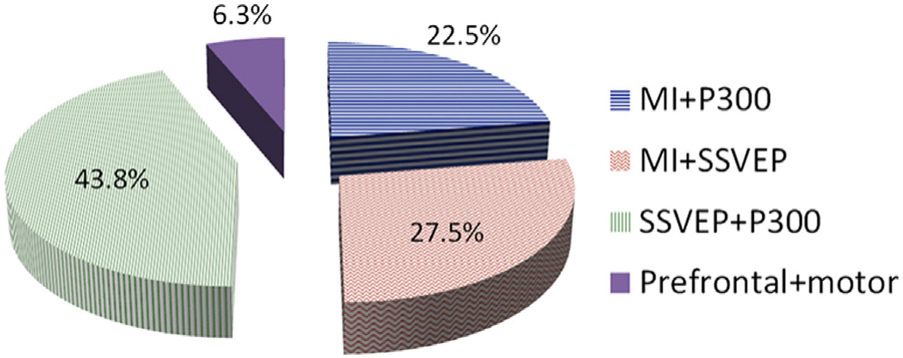
Hybrid brain–computer interface paradigms combining different brain signals (2009–2016).

Most of the work on brain activity combination hBCIs has been based on SSVEP- and P300-based paradigms. Although both are reactive tasks, the most widely observed applications have been in the area of speller and wheelchair control. In relation to the combination of MI with P300 or SSVEP, the applications are widely used in neuro-rehabilitation and control settings. Only a very small portion of hBCI research has targeted prefrontal and motor-based hBCI. This strategy is useful in increasing the number of commands for both fNIRS alone and combined EEG–fNIRS. A breakdown of the paradigms employed between 2009 and 2016 is shown in Figure 8.

The hBCI can enhance the classification accuracy and increase the number of commands of a BCI system without influencing either of those two factors. The trend in hBCI (see Figure 6) suggests the high potential of research in this field. Although the early BCI (i.e., single modality) problems were dealt with by combining modalities, there are still several research issues that remain untouched.

**Figure 9:**
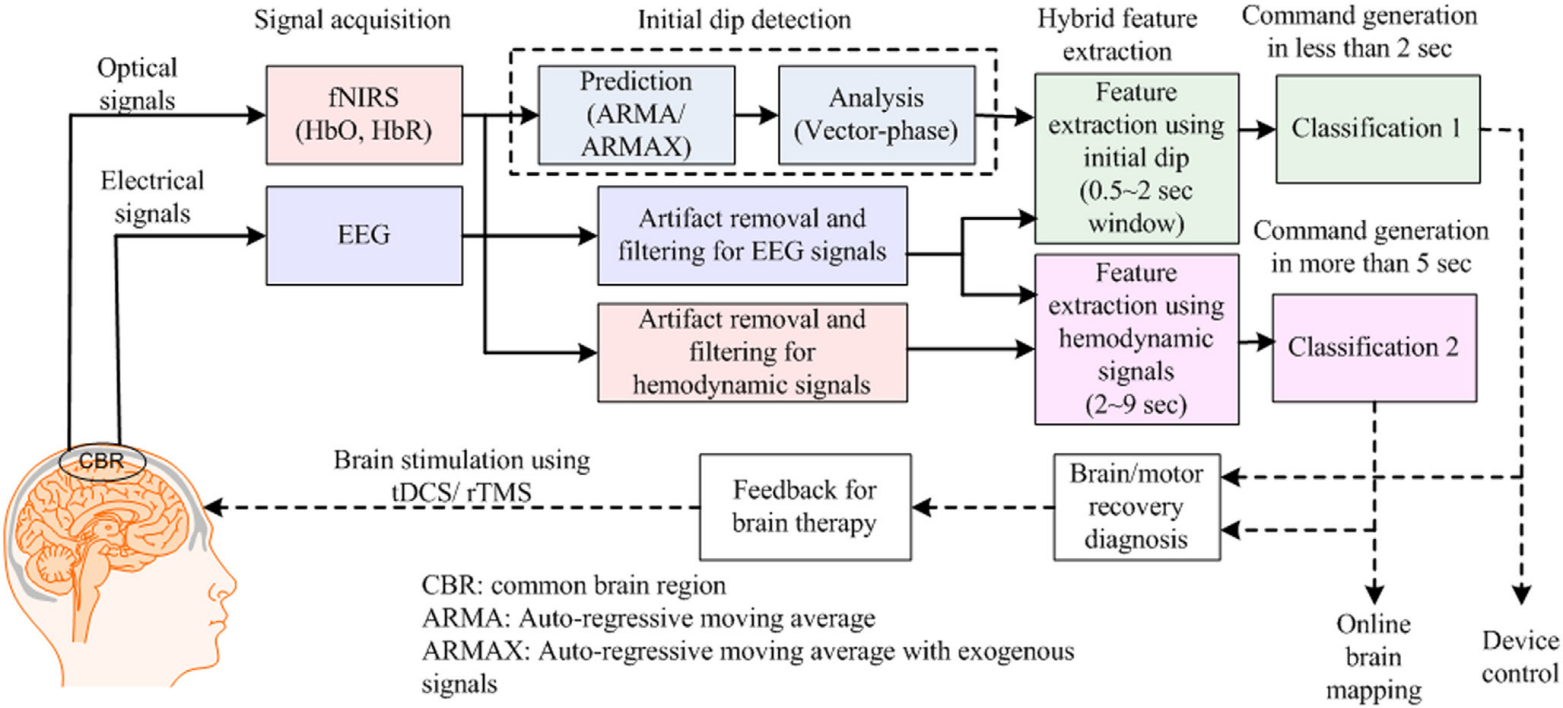
The proposed hybrid electroencephalography (EEG)–NIRS using hemodynamic and initial dip features for simultaneous activity detection and classification.

Although window smoothing techniques are available in literature (Qi et al., 2012), one of the most important questions in the development of hBCIs is the selection of window size. Several researchers have worked on the problem of an optimal window size for BCI; however, the literature still lacks any conclusive work on standardized window selection for simultaneous decoding of brain activates. In hybrid systems, different windows that were optimized for individual modalities are naturally used for feature extraction (Ma et al., 2015; Buccino et al., 2016; Khan and Hong, 2017). This will result in a delay in making a final decision until the data from a bigger window are processed. Therefore, a new decision making scheme suitable for hybrid systems needs to be developed. To the best of the authors’ knowledge, an algorithm that can simultaneously extract/classify features even for simple EEG–fNIRS dual modalities applied to the same brain area has not been developed yet. Especially, in the case of combined EEG–fNIRS, the reported optimal window size is 10 s (Tomita et al., 2014), which might not be appropriate for the control of external devices. Further improvement should be achieved, for example, by using initial dip detection (Jasdzewski et al., 2003; Yoshino and Kato, 2012; Hong and Naseer, 2016) instead of relying on the hemodynamic response of fNIRS together with EEG signals in the reduction of window size. For this particular purpose, a recent study has shown the feasibility of initial dip detection for application to BCI (Hong and Naseer, 2016). In terms of classification, two studies (Khan and Hong, 2017; Zafar and Hong, 2017) have reported the classification of prefrontal fNIRS signals within a 2 s window. However, in those studies, the EEG and fNIRS signals were not simultaneously decoded for a single task but recorded from two different tasks. A significant amount of research is needed to decode EEG–fNIRS signals simultaneously for enhanced accuracy without influencing the EEG signal detection time. Developments in this area can produce fruitful results by which the hybrid window size can be reduced (less than 1 s). The use of a fast optical response (Hu et al., 2011), furthermore, also could help to reduce the window size. Perhaps the use of multi-wavelength system (Bhutta et al., 2014) combined with adaptive signal processing algorithms (Hu et al., 2010, 2013; Santosa et al., 2013; Ren et al., 2014; Zhang et al., 2016b; Zhou et al., 2016) will significantly contribute to the eventual reduction of the inherent delay in the hemodynamic response. Figure 9 shows the proposed hybrid EEG–fNIRS model for window reduction.

Another important aspect that requires a focus with respect to hBCI is the selection of active control commands. The reactive commands can be increased by changing the flickering stimuli for BCI. In fact, using reactive tasks, more than 50 commands can be achieved (see Table 4). A BCI using active commands is more desirable than one based on reactive commands. After, at most, three or four active commands, the accuracy severely drops, making it difficult to control an external device with a further increased number of commands. The current need is such strategies that can be used to achieve active control of BCI systems without impacting negatively on accuracy. In this regard, the hBCI can play an important role. Future research in this area will provide a solution to the problems related to the increase in the number of active commands.

Besides the issues of the number of commands, classification accuracy, and detection time, the following challenging issues need to be investigated: (i) how to predict the desired feature from a slow-modality signal in synchronizing the classification time to a fast-modality signal, (ii) development of a general meta-feature model covering the multiple modalities considered, (iii) development of multiple interactive models switching based on their computed probabilities, (iv) optimization of a brain region for hybrid modalities, (v) optimization of the number of sensors (i.e., electrodes and optodes) needed for BCI, (vii) finding of the best combination of local brain regions for hybrid imaging, (viii) how to synchronize brain and non-brain signals if the hybridization is extended beyond the brain, (ix) how to fuse multiple information to single out one definitive decision, and (x) how to deliver the information obtained from one modality to others.

The current need is the development of a portable, wearable, and low-cost hBCI system that can be used for both healthy persons and patients. Furthermore, motion artifacts should be minimized, and there should be the capacity to enhance accuracy and increase the number of commands as needed. Moreover, the hBCI should be designed from the application point of view. Currently, however, no such hBCI systems are commercially available. If such system exists, its combination with a haptic device (Nam et al., 2014, 2015) may provide better assistance to patients in movement and sensing. Whereas EMG/EOG combined with EEG can be used for control applications (e.g., wheelchair control), the most significant breakthrough in hBCI is the design of hybrid EEG–NIRS that can simultaneously decode electrical and hemodynamic brain activities. Considering the fact that fMRI has high spatial but low temporal resolution, further research in hybrid EEG–NIRS might be a more promising brain-diagnostic endeavor. In the near future, this can be made possible with a breakthrough by combining real-time EEG rhythmic cortical activity monitoring (Im et al., 2007) with fNIRS directional coupling estimation (Im et al., 2010) and bundled optodes based 3D imaging techniques (Nguyen and Hong, 2016; Nguyen et al., 2016). Such advances, enabling the utilization of non-invasive methods, will allow for a much better understanding of the brain.

## Conclusion

In this article, we have reviewed the state-of-the-art research on hBCI technologies. We have discussed the hardware and methodologies adopted by researchers for the development of the pertinent hBCI systems. The most recent work related to the hardware combinations and strategies adopted for several brain signal acquisition modalities have been discussed as well.

The issue of hBCI hardware is addressed in light of the employed combinations of EEG with fNIRS, EOG, and EMG. EEG and fNIRS are combined to enhance classification accuracy and to increase the number of control commands for BCI systems. The brain activity features are combined and simultaneously decoded to improve the BCI performance. The combination of EEG with EOG has a similar end, EOG being used to increase the number of commands or to remove motion artifacts for an improved accuracy. EMG, meanwhile, is used to remove motion artifacts and, thereby, improve the classification accuracy.

Multi-modality improves classification accuracy and increases the number of control commands: the number of commands can be increased by simultaneously decoding the brain activities in hybrid paradigms. In this case, we have discussed the increase in the number of commands using steady-state visual evoked potentials (SSVEP) and ERP. Also, the combination of motor and prefrontal tasks for the development of hBCI paradigms has been discussed.

Although the hBCI issues span both the increase in the number of active commands and the improvement in classification accuracy, some additional concerns remain: the selection of optimal features and windows for activity detection, for example, is still relatively neglected. In any case, it is clear that there is much room for future hBCI research, particularly, in its applications. The field is still young. For example, there is as yet no commercially available hBCI system, notwithstanding the several communication and control strategies that have already been introduced. Doubtless several control and rehabilitation application breakthroughs are at hand.

## Author Contributions

KSH conceived the topic. MJK conducted the literature survey and wrote a preliminary version. KSH finalized the paper.

## Conflict of Interest Statement

The authors declare that the research was conducted in the absence of any commercial or financial relationship that could be construed as a potential conflict of interest.

## Abbreviations

BCI, brain–computer interface; CCA, canonical correlation analysis; CP, cerebral palsy; EEG, electroencephalography; EOG, electrooculography; EMG, electromyography; ERD, event-related desynchronization; ERP, event-related potentials; FDA, Fisher discriminant analysis; FLDA, Fisher LDA; FES, functional electrical stimulation; fMRI, functional magnetic resonance imaging; fNIRS, functional near infrared spectroscopy; GA, genetic algorithm; GUI, graphical user interface; hBCI, hybrid brain–computer interface; HbO, oxyhemoglobin; HbR, deoxyhemoglobin; HMM, Hidden Markov model; ICA, independent component analysis; LDA, linear discriminant analysis; LIS, locked-in syndrome; MA, mental arithmetic; MCS, minimally conscious state; MI, motor imagery; PSO, particle swarm optimization; SCI, spinal cord injury; SM, signal mean; SMR, sensory motor rhythm; SS, signal slope; SSSEP, steady-state somatosensory evoked potentials; SSVEP, steady-state evoked visual potentials; SVM, support vector machine; VS, vegetative state; SW-LDA, step-wise LDA; tDCS, transcranial direct current stimulation; rTMS, repetitive transcranial magnetic stimulation.
